# Climbing Up and Down Binding Landscapes through Deep Mutational Scanning of Three Homologous Protein–Protein Complexes

**DOI:** 10.1021/jacs.1c08707

**Published:** 2021-10-05

**Authors:** Michael Heyne, Jason Shirian, Itay Cohen, Yoav Peleg, Evette S. Radisky, Niv Papo, Julia M. Shifman

**Affiliations:** † Department of Biological Chemistry, The Alexander Silberman Institute of Life Sciences, The Hebrew University of Jerusalem, Jerusalem, 9190401, Israel; ‡ Avram and Stella Goldstein-Goren Department of Biotechnology Engineering and the National Institute of Biotechnology in the Negev, Ben-Gurion University of the Negev, Beer-Sheva, 8410501, Israel; § Life Sciences Core Facilities (LSCF) Structural Proteomics Unit (SPU), Weizmann Institute of Science, Rehovot, 7610001, Israel; ∥ Department of Cancer Biology, Mayo Clinic Comprehensive Cancer Center, Jacksonville, Florida 32224, United States

## Abstract

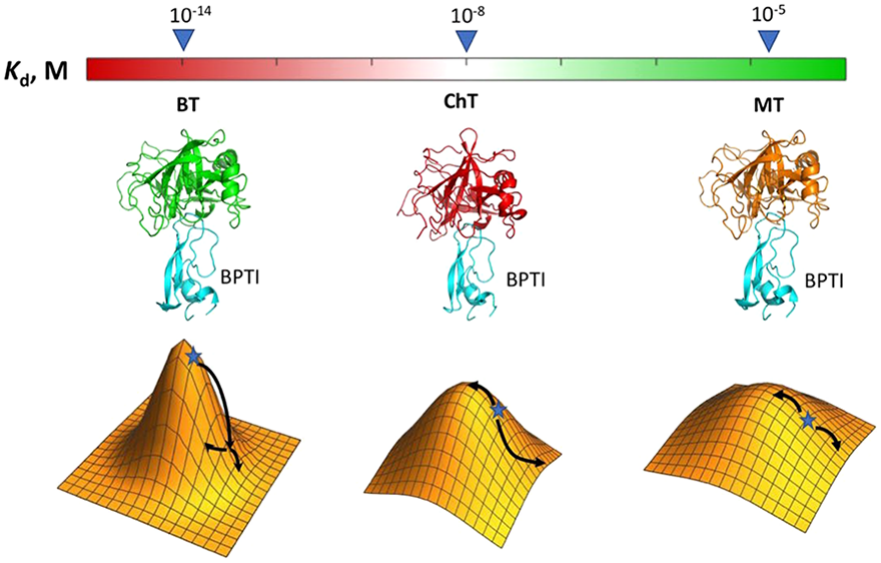

Protein–protein interactions (PPIs) have evolved to display binding affinities that can support their function. As such, cognate and noncognate PPIs could be highly similar structurally but exhibit huge differences in binding affinities. To understand this phenomenon, we study three homologous protease–inhibitor PPIs that span 9 orders of magnitude in binding affinity. Using state-of-the-art methodology that combines protein randomization, affinity sorting, deep sequencing, and data normalization, we report quantitative binding landscapes consisting of ΔΔ*G*_bind_ values for the three PPIs, gleaned from tens of thousands of single and double mutations. We show that binding landscapes of the three complexes are strikingly different and depend on the PPI evolutionary optimality. We observe different patterns of couplings between mutations for the three PPIs with negative and positive epistasis appearing most frequently at hot-spot and cold-spot positions, respectively. The evolutionary trends observed here are likely to be universal to other biological complexes in the cell.

## Introduction

Protein function is determined by the protein amino acid sequence, which has undergone billions of years of evolution while subjected to various selection pressures. Native proteins have evolved not only to perform their main function but also to satisfy a number of criteria such as solubility,^1^ low propensity for aggregation, stability, resistance to stress conditions,^2^ etc. As a result of these opposing pressures^3,4^ and mutation-selection balance,^5^ proteins usually function below their maximum capacity.^6,7^ Multiple experiments on enzymes and binding domains proved that protein fitness could be enhanced by several orders of magnitude by applying an appropriate pressure and selecting the fittest protein sequences.^8−11^

Fitness landscapes explore the effects of all possible mutations on the ability of proteins to perform their main function. Such landscapes reveal how far a particular protein is from its functional maximum, what fraction of mutations leads up and down the “fitness hill”, how large the mutational steps are, and which residues are the most critical to protein function.^12^ Mapping of fitness landscapes is thus an attractive strategy for approaching various protein engineering projects with the goal to improve or modify protein function since the best mutations could be easily identified from the fitness landscape.^13,14^ Development of new strategies for protein randomization and advances in next-generation sequencing (NGS) enabled several exciting studies that report fitness landscapes for a number of biological systems.^1,15−27^ In these studies, the effects of mutations on enzyme catalysis, fluorescence, thermostability, and other functions have been reported, giving invaluable insights on how different biological functions have evolved.

Binding between two or more protein partners represents one of many important protein functions. Binding is crucial in many cellular activities such as signal transduction, protein regulation, transcription, translation, and others. Mutations in protein–protein interactions (PPIs) frequently result in a change in free energy of binding (ΔΔ*G*_bind_), sometimes weakening and sometimes stabilizing the interaction.^28^ A mutation resulting in substantial ΔΔ*G*_bind_ in one PPI could translate into remodeling of the whole PPI network, frequently leading to dysregulation of signal transduction pathways and disease.^29,30^ Therefore, understanding how mutations in PPIs affect their binding affinity is of great importance to both basic biology and to biomedical sciences, where inhibition or activation of a particular PPI might help to treat the related disease.

At the present moment, comprehensive binding landscapes have been mapped for only a handful of proteins,^13,31−36^ while for most PPIs only a few ΔΔ*G*_bind_ data points have been measured, most frequently involving mutations to alanines.^37−41^ Comparison of the available sparse ΔΔ*G*_bind_ data from different studies led us to hypothesize that different classes of PPIs possess principally different binding landscapes and lie at different points relative to the binding landscape maximum, i.e., the amino acid sequence with the highest possible affinity. While in some PPIs, the majority of single mutations lead to large destabilization of the protein–protein complex,^33,42−44^ in other PPIs frequent affinity-enhancing mutations are observed.^32,45^ The magnitude of ΔΔ*G*_bind_ due to mutation is likely to depend on the nature of the PPI under study as well as on the location of the mutation within the protein. It has been demonstrated that a few critical positions, termed hot-spots of binding, contribute the most significantly to the PPI binding energy with mutations at those positions usually leading to a large reduction in affinity.^46−48^ Cold-spot positions, on the other hand, present multiple possibilities for PPI affinity improvement.^49^

**Figure 1:**
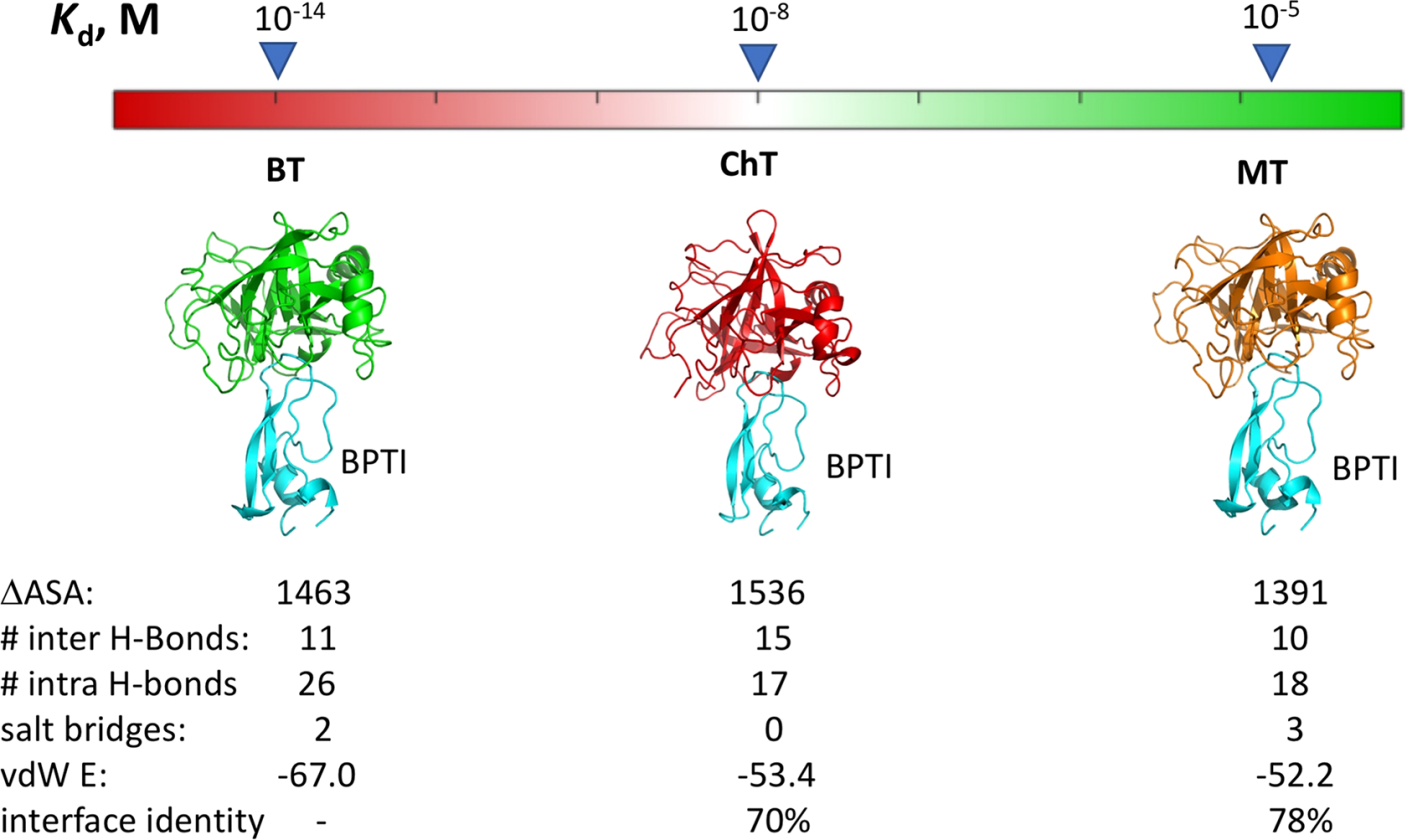
Comparison of *K*_D_s and structures for the three PPIs. Structures of the three complexes between BPTI and BT (PDB ID 3OTJ), ChT (PDB ID 1CBW), and MT (PDB ID 2R9P) are shown. Summarized are inhibition constants, change in solvent-accessible surface area upon complex formation in A^2^ (ΔASA), number of intra- and inter-molecular hydrogen bonds, salt bridges, van der Waals (vdW) energy, and interface sequence identity of proteases relative to BT.

To investigate the basis for large differences in binding affinity between evolutionarily optimized cognate PPIs and nonoptimized noncognate PPIs, we compare comprehensive binding landscapes of three structurally similar PPIs that span 9 orders of magnitude in binding affinity (*K*_D_) (Figure 1). We examine the interaction of bovine pancreatic trypsin inhibitor (BPTI) with its coevolved biological target bovine trypsin (BT) (*K*_D_ = 10^–14^ M)^44^ and with noncognate trypsin paralogs bovine α-chymotrypsin (ChT) (*K*_D_ = 10^–8^ M)^44^ and human mesotrypsin (MT) (*K*_D_ = 10^–5^ M).^50^ BPTI has coevolved with trypsin to protect the pancreas from premature trypsin activation and consequent autodigestion of the organ. BPTI is a compact 58-amino acid protein with three disulfide bonds; it binds to BT by inserting a binding loop into the trypsin active site in a substrate-like manner, with a Lys residue occupying the complementary trypsin specificity pocket.^51^ The backbone of the BPTI binding loop is preconfigured in canonical conformation for lock-and-key recognition at the BT active site, forming main chain–main chain hydrogen bonds between inhibitor and enzyme that are structurally conserved across complexes with different proteases.^51,52^ The structure of BPTI thus acts as a “molecular vise”,^42^ forcing the same Lys residue into the specificity pocket of most BT paralogs, irrespective of whether the residue forms favorable or locally deleterious interactions. Consequently, BPTI forms a structurally similar although weaker PPI with ChT, which possesses specificity for cleavage after large hydrophobic residues rather than Lys/Arg. BPTI likewise forms a similar PPI with MT, a trypsin paralog that is present only in hominids, having arisen from a relatively recent gene duplication.^53^ MT retains the primary specificity of other trypsins for cleavage after Lys/Arg but has evolved unique resistance to inhibition by canonical trypsin inhibitors due to several mutations near the active site, resulting in much weaker affinity toward BPTI.^54−56^ Consistent with the structural homology between these three PPIs, they exhibit binding interfaces of nearly identical physicochemical properties despite their large differences in *K*_D_ values (Figure 1).

In an attempt to better understand the drastic differences in binding affinities between these PPIs, we explored ΔΔ*G*_bind_ values between the three proteases and all single and double binding interface mutants of BPTI. To measure ΔΔ*G*_bind_ values for tens of thousands of mutants in these three PPIs, we employed a strategy recently developed by our group that relies on protein randomization, yeast surface display (YSD) technology, NGS analysis, and a small experimental data set of ΔΔ*G*_bind_ values measured using purified proteins to generate ΔΔ*G*_bind_ values for the remaining numerous mutants of the same protein complex.^57^ We previously demonstrated that the above method produces a very high correlation (*R* > 0.9) between the NGS-based predictions of ΔΔ*G*_bind_ values and the actual experimental values.^57^ In the present study, we use this state-of-the-art methodology to construct and compare binding landscapes of the three structurally similar BPTI/protease complexes.

Our data demonstrate that the three complexes possess drastically different binding landscapes and lie at different points with respect to the binding landscape maximum. Additionally, these differences in landscape contour and placement underlie correspondingly different energetic consequences of mutation, including asymmetrical directionality and different tendencies toward positive or negative epistasis.

## Results

**Figure 2:**
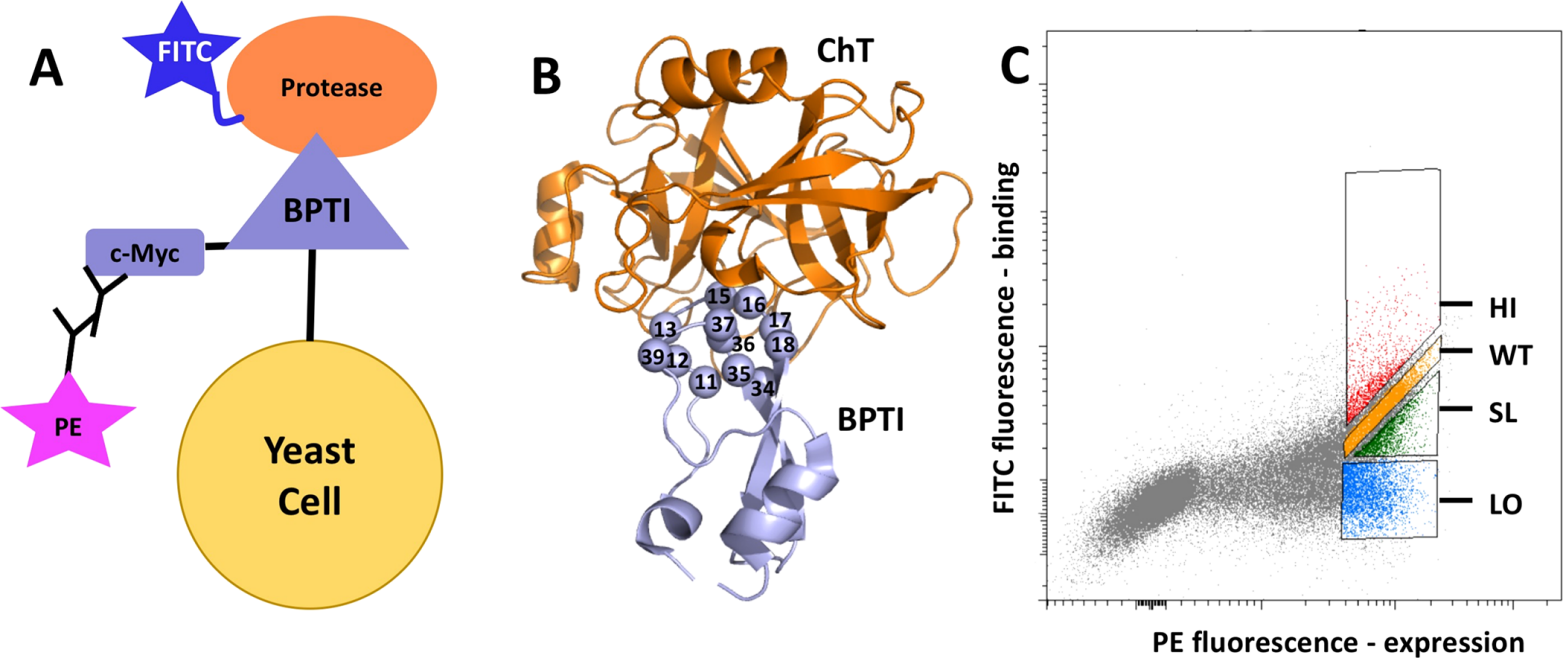
(A) Yeast surface display construct with BPTI displayed on the surface of yeast cells. C-Myc tag is used to monitor BPTI mutant expression using PE-labeled antibody. Proteases are labeled by FITC that is used to monitor binding between the two proteins. (B) Construction of the BPTI mutant library. Structure of the ChT/BPTI complex with ChT shown in orange and BPTI in violet. BPTI binding interface positions randomized to 20 amino acids are shown as spheres. (C) FACS data showing sorting of BPTI mutants binding to protease into four different populations. The uppermost HI gate contains BPTI mutants with affinity higher than that of BPTI_WT_. The second uppermost gate, WT, contains BPTI mutants with an affinity similar to BPTI_WT_. The third gate, SL, contains BPTI mutants with an affinity slightly lower than that of BPTI_WT_, and the lowest gate, LO, contains BPTI mutants with an affinity much lower than that of BPTI_WT_. The data are shown for the BPTI/ChT interaction, while similar data were obtained for the BPTI/MT and BPTI/BT interactions.

To map binding landscapes of the three homologous BPTI/protease complexes, we first incorporated the wild-type BPTI (BPTI_WT_) gene into the pCTCON vector, compatible with YSD technology. Using this construct, BPTI_WT_ was expressed on the surface of a yeast cell with a C-terminal myc-tag for monitoring protein expression through binding of an antimyc antibody and a secondary antibody conjugated to phycoerythrin (PE) (Figure 2A). Binding of a protease to BPTI_WT_ was accessed by monitoring fluorescence of the FITC fluorophore conjugated to the protease via neutravidin. The assessment of binding of BPTI_WT_ to the three proteases by fluorescence-activated cell sorting (FACS) showed a diagonal narrow distribution, demonstrating that BPTI_WT_ is well expressed on the surface of yeast cells, is properly folded, and binds to each of the proteases (Figure S1 in the Supporting Information).

We next generated a library of BPTI mutants that contained all single and double BPTI mutants at positions that comprise the direct binding interface with proteases in the BPTI/protease structures. We randomized 12 BPTI positions to 20 amino acids while leaving two cysteines that participate in a disulfide bond intact to preserve BPTI folding (Figure 2B). In addition, all possible combinations of double mutations encompassing these 12 positions were encoded in the library. The BPTI library, referred to as the naïve library, contained 228 single mutants and all possible pairs of such mutations, resulting in the total theoretical diversity of 26,400 BPTI sequences. The naïve library was transformed into yeast and sequenced by NGS. Sequencing results showed that all possible single mutations were covered in the naïve library. Among double mutants, we saw 89% of all possible sequences; this percentage was reduced to 60% when a cutoff of five sequencing reads was applied.

**Figure 3:**
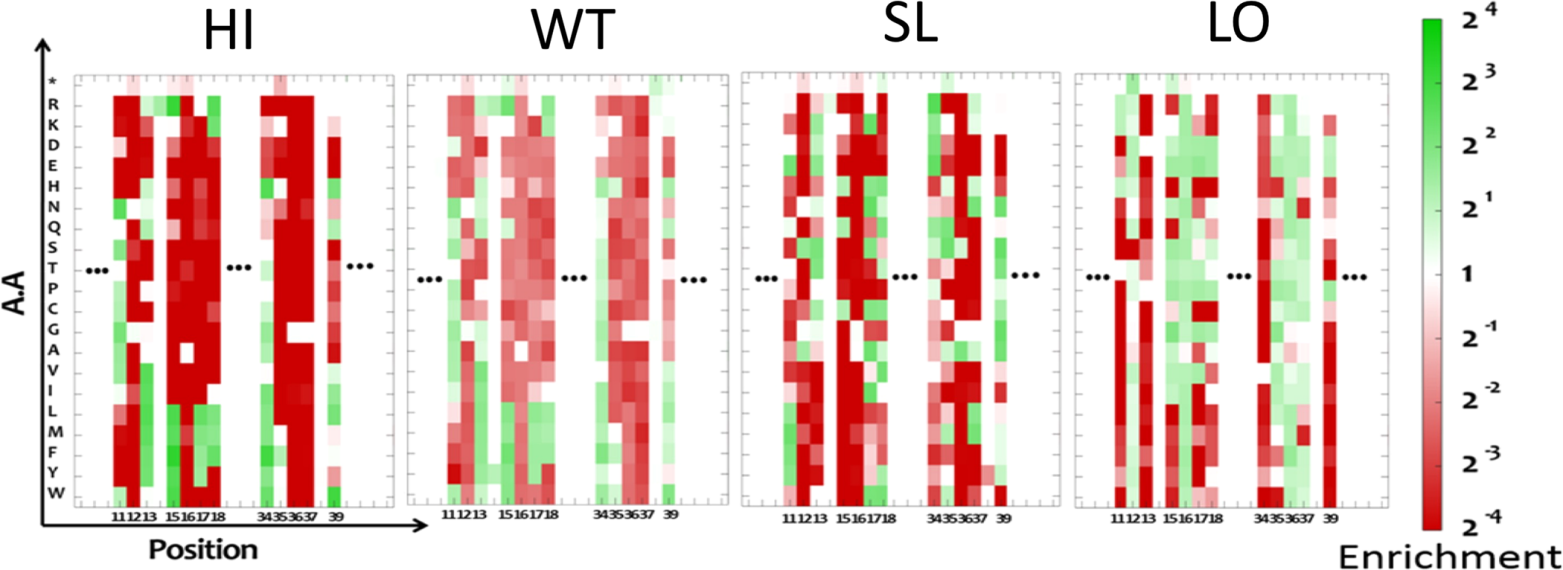
Heatmap showing the enrichment values for each single mutation in the ChT/BPTI complex in the four sorted affinity gates. The enrichment ratio varies from high (green) to low (red), as shown on the right axis. Similar maps were obtained for the other two PPIs.

We next expressed the BPTI library on the yeast surface and measured expression and binding of the BPTI library to the three proteases using FACS (Figure 2C). The concentration of each protease was optimized to exhibit a considerable spread of the FACS binding signals from different BPTI mutants (Figure S2 in the Supporting Information). For each protease, we performed a sorting experiment and collected yeast cells with BPTI mutants belonging to four different affinity groups: higher than WT affinity (HI), WT-like affinity (WT), slightly lower than WT affinity (SL), and strongly lower than WT affinity (LO) (Figure 2C and Figures S3–S5 in the Supporting Information). The cells from each affinity gate were grown and sequenced with NGS, resulting in 300–900 K reads per each population. For each BPTI mutant and each protease, we next calculated the enrichment value, which represents the ratio between the mutant’s frequency in a particular affinity gate to its frequency in the naïve library. We thereby obtained heatmaps of the enrichment values for all positions as shown in Figure 3 for the CT/BPTI interaction.

While the enrichment maps give us qualitative measures of affinity changes due to various mutations, our goal was to construct and compare quantitative binding landscapes of the BPTI/protease interactions. We thus utilized the methodology developed in our recent paper that allows us to normalize the NGS-based enrichments using a small data set of experimental ΔΔ*G*_bind_ values measured by biophysical techniques on purified proteins.^57^ We first compiled such normalization data sets for the three complexes, collecting 34 and 33 ΔΔ*G*_bind_ data points from the literature for the ChT/BPTI and BT/BPTI interactions, respectively.^44,57−60^ For the MT/BPTI interaction, where only a few ΔΔ*G*_bind_ data points have been reported,^50,61^ we produced the normalization data set by expressing and purifying 12 BPTI mutants and measuring their binding affinities to MT (Figure S6 in the Supporting Information). The above data sets were used to obtain a normalization formula for each protease that converts the four enrichment values from the NGS data into the predicted ΔΔ*G*_bind_ values. For all three enzymes, high correlation was found between the ΔΔ*G*_bind_ values predicted from NGS and those experimentally determined using purified proteins (*R* ≈ 0.9; Figure S7 in the Supporting Information).

**Figure 4:**
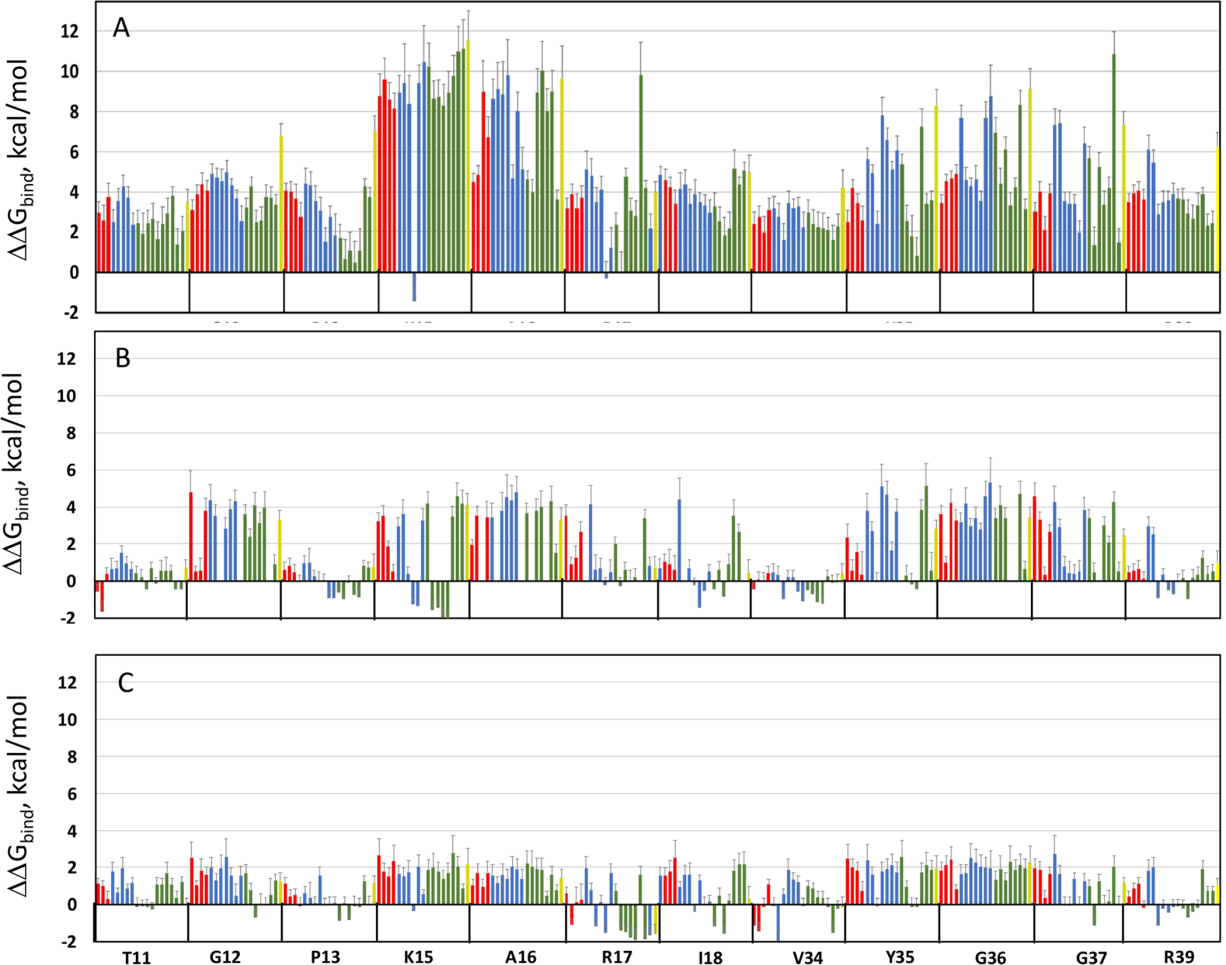
Changes in ΔΔG_bind_ for all single mutants of BPTI interacting with BT (A), ChT (B), and MT (C). Each bar represents a mutation to one amino acid including hydrophobic amino acids (green), polar amino acids (red), charged amino acids (blue), and Cys (yellow). The *x*-axis shows the WT residue followed by position. Error bars represent the 95% CI. Figure (A) in reprinted without changes from Heyne, M.; Papo, N.; Shifman, J. M. Generating quantitative binding landscapes through fractional binding selections combined with deep sequencing and data normalization. *Nat. Commun.***2020**, *11* (1), 1–7, under the terms of the CC BY license (Creative Commons Attribution 4.0 International License), Copyright © 2020, The Author(s).

We next used these normalization formulas to predict ΔΔ*G*_bind_ values for all single and double BPTI mutants that were detected by NGS for the three PPIs. While nearly all BPTI single mutants were sequenced in all four affinity gates for the three proteases, the double mutants were covered less extensively in the NGS results with only 576, 3393, and 636 double mutants appearing in all four affinity gates for ChT, BT, and MT, respectively. The relatively low coverage of double mutants is due to two main reasons. First, some BPTI mutants exhibited low folding stability, resulting in their low expression on yeast. Such destabilized mutants were not collected in our selection experiments for all three PPIs. Second, the number of NGS runs was not sufficient to detect all ∼26 000 double mutants in all affinity gates, thus resulting in some differences in invisible mutants for each protease. To increase the coverage of ΔΔ*G*_bind_ predictions for the double mutants and to complete the predictions for single mutants, we examined whether normalization formulas could be obtained from subsets of three, two, and one affinity gate. While all subsets of gates were examined, only those subsets that produced high correlation with experimental data on pure proteins were selected for the final predictions. For each ΔΔ*G*_bind_ prediction, we estimated the uncertainty in ΔΔ*G*_bind_ predictions using the bootstrapping of the NGS data (see Methods for details). Overall, we were able to make reliable predictions for 13 113 double mutants for the BT/BPTI interaction (50% of all binding interface double mutations), 12 537 for the ChT/BPTI interaction (47%), and 13 354 for the MT/BPTI interaction (51%). We thus constructed full single mutant binding landscapes (Figure 4) and partial double mutant binding landscapes for BPTI interacting with the three homologous proteases with highly divergent *K*_D_s.

### Analysis of the Single Mutant Binding Landscapes

**Figure 5:**
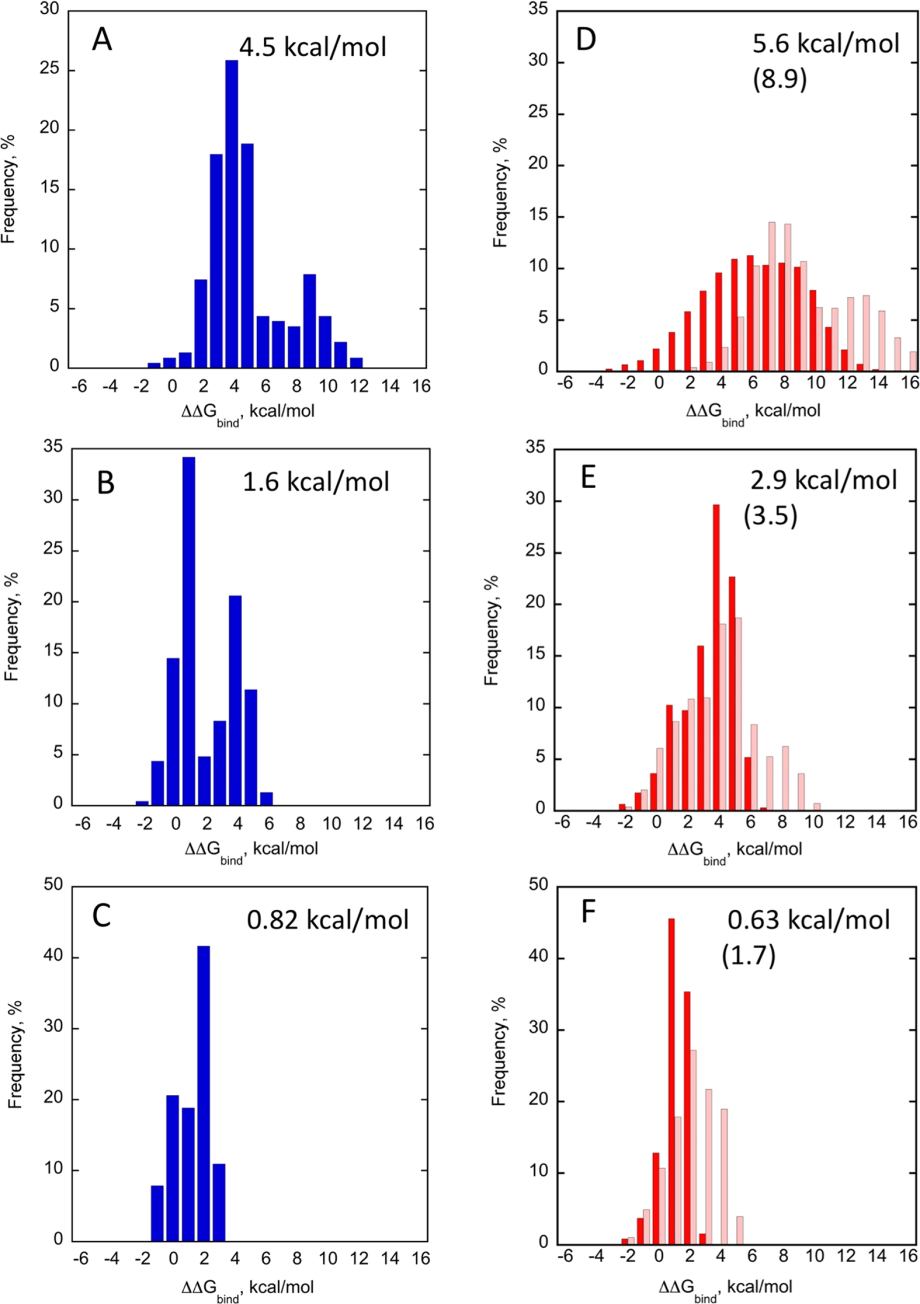
Histograms of ΔΔ*G*_bind_ for single and double BPTI mutants. Single BPTI mutants interacting with (A) BT; (B) ChT; and (C) MT. Double BPTI mutants interacting with (D) BT; (E) ChT; and (F) MT. Dark red represents the actual measured values, while light red represents the values that would result from all single mutations being additive. Mean value for ΔΔ*G*_bind_ for each histogram is displayed on top of each graph; in parentheses is the value that would result from all double mutations being additive. While all 228 single mutants are incorporated into the single mutant histograms for all proteases, only ∼50% of double mutants are summarized. The data are available in the Source Data file in the Supporting Information.

**Figure 6:**
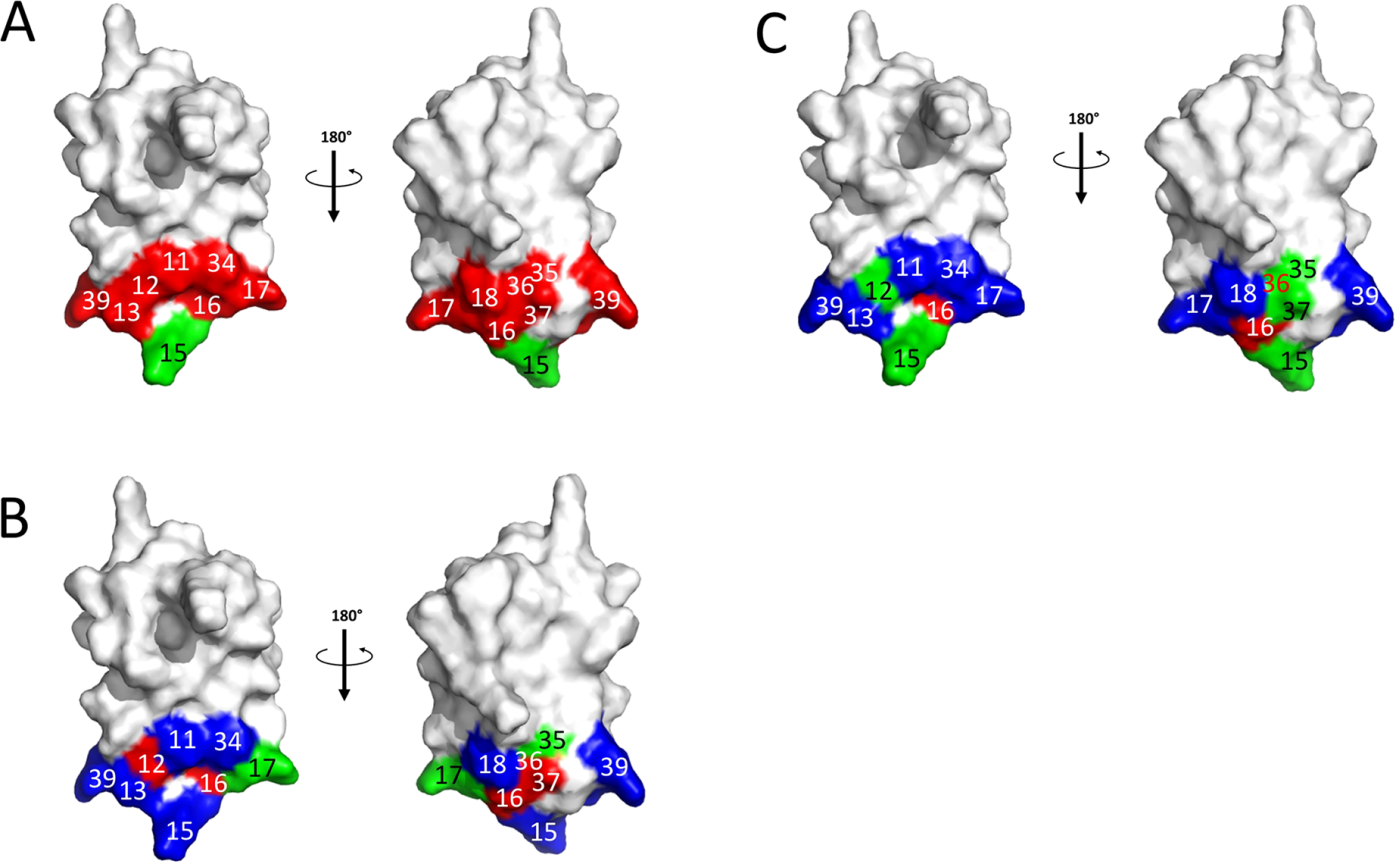
Structure of BPTI with binding interface positions colored according to the number of detected affinity-enhancing mutations at this position when interacting with (A) BT; (B) ChT; and (C) MT. Red: no affinity-enhancing mutation was detected; green: 1 or 2 affinity-enhancing mutations were detected; blue: 3 or more affinity-enhancing mutations were detected.

To compare how single mutations affect free energy of binding in the three PPIs, we summarized our results in a histogram that includes ΔΔ*G*_bind_ values from all 228 single mutations for each PPI (Figure 5A–C). While all three histograms show predominance of destabilizing mutations (ΔΔ*G*_bind_ > 0), the magnitude of destabilization due to single mutations differs substantially among the three PPIs. For the high-affinity BT/BPTI complex, very high ∼12 kcal/mol destabilizations were observed due to some single mutations, medium destabilizations (up to 6 kcal/mol) were observed in the ChT/BPTI complex, and small destabilizations (up to ∼3 kcal/mol) were observed for the low-affinity MT/BPTI complex (Figure 4 and Figure 5A–C). On average, a single mutation destabilized a BT/BPTI interaction by 4.5 kcal/mol, a ChT/BPTI interaction by 1.6 kcal/mol, and an MT/BPTI interaction by 0.82 kcal/mol. Affinity-enhancing mutations appeared more frequently in the low-affinity MT/BPTI complex (50 mutations or 22%), less frequently in the medium-affinity ChT/BPTI complex (37 mutations or 16%), and only once (<1%) in the high-affinity BT/BPTI complex. Per-position analysis of ΔΔ*G*_bind_ values revealed that all but one position on BPTI were absolute hot-spots in the BT/BPTI interaction, exhibiting only positive ΔΔ*G*_bind_ values (Figure 4). In contrast, only four absolute hot-spots were present in the ChT/BPTI interaction (positions 12, 16, 36, 37) and only two in the MT/BPTI interaction (position 16 and 36). The spatial distribution of cold-spot and hot-spot positions showed different patterns among the three complexes (Figure 6). Position 15 is nearly a hot-spot in both the BT/BPTI and the MT/BPTI complexes, with all mutations leading to high destabilization of the two complexes except for the K15R mutation, which leads to affinity improvement. However, the same position is a cold-spot in the ChT/BPTI complex, where all hydrophobic amino acids lead to affinity improvement. These differences in the position 15 preferences are in complete agreement with previous studies on purified proteins for the BT/BPTI and ChT/BPTI complexes.^59^ Additionally, the amino acid preferences of BPTI at position 15 observed here reflect the preferences for substrates that these enzymes cleave (Lys and Arg for trypsins and hydrophobic amino acids for chymotrypsins), indicating that these enzymes have evolved to possess optimal binding pockets for these amino acids.

### Analysis of the Double Mutant Binding Landscapes

We next compared the double mutant binding landscapes for the three PPIs. We first plotted the histograms of ΔΔ*G*_bind_ values for ∼50% of all double mutations, for which ΔΔ*G*_bind_ predictions were available (Figure 5D–F). Our results show that, on average, a double mutation destabilizes the high-affinity BT/BPTI complex by 5.9 kcal/mol, the medium affinity ChT/BPTI complex by 2.9 kcal/mol, and the low-affinity MT/BPTI complex by 0.63 kcal/mol, showing the same tendency of increased destabilization due to double mutation with increasing affinity of the PPI as observed for single mutants. When comparing an average effect from a double and a single mutation, BT/BPTI and ChT/BPTI exhibit a higher mean ΔΔ*G*_bind_ value for a double mutation, consistent with the interpretation that a majority of single mutations are destabilizing in these two PPIs. For the low-affinity MT/BPTI complex, the double mutant average is slightly lower compared to that of the single mutant average, consistent with the fact that in this PPI many affinity-enhancing mutations have been detected. All three double mutation distributions exhibit a lower mean ΔΔ*G*_bind_ value in comparison to what would be predicted from additivity of all single mutations (Figure 5D–F). These results could be explained by the fact that ∼50% of the BPTI double mutations are absent from our analysis. Among the invisible mutations, the majority result in high BPTI destabilization and are likely to exhibit high positive ΔΔ*G*_bind_ upon binding to proteases. Such mutations, if included in the analysis, would shift the double mutant distribution to higher mean ΔΔ*G*_bind_ values for all three PPIs. The minority of the double mutants are missing due to their low coverage in the NGS data. These mutants, however, are randomly distributed in the ΔΔ*G*_bind_ histogram and are not expected to change the mean.

Using the extensive ΔΔ*G*_bind_ data for double mutations, we further explored how a single mutational step from a WT sequence alters the distribution of ΔΔ*G*_bind_ values for the second mutation. For this analysis, we selected three representative single BPTI mutants in the cognate BPTI/BT complex: BPTI_K15R, which shows slight improvement in affinity compared to BPTI_WT_ (ΔΔ*G*_bind_ = −1.4 kcal/mol), BPTI_A16S, whose affinity to BT is considerably weaker in comparison to BPTI_WT_ (ΔΔ*G*_bind_ = +4.5 kcal/mol), and BPTI_K15A, which shows dramatically reduced affinity in comparison to BPTI_WT_ (ΔΔ*G*_bind_ = +11.1 kcal/mol). We next compared the ΔΔ*G*_bind_ distributions for single mutations taken on the background of each of the three specified first mutations. While only partial single mutant landscapes could be constructed for these three BPTI mutants interacting with BT (as we have the data for ∼50% of the double mutants), for the detected mutants we observe significant differences in the binding landscapes of the three BPTI mutants with BT (Figure S8 in the Supporting Information). K15R, which improves the affinity of the BT/BPTI interaction, produces a histogram with mostly destabilizing mutations going as far as +12 kcal/mol, yet some affinity-enhancing mutations are also observed. The medium-destabilizing mutation A16S results in a distribution that contains both stabilizing and destabilizing steps with magnitudes ranging from −6 to +8 kcal/mol. The highly destabilizing mutation K15A exhibits a distribution that mostly contains stabilizing mutations with the highest stabilization of −6 kcal/mol. Note that for the K15A mutant we do not observe a mutational step that would reach the affinity of the WT BT/BPTI complex. This is likely because position 15 is the most important energetically for the BPTI/protease interaction; thus destroying the favorable interaction at this position could not be fully compensated by any other mutation on BPTI. Our results hence indicate that with every mutational step taken from the WT BPTI sequence the binding landscape would be changed depending on the first mutation; this change is a result of nonadditivity of some of the single mutations in BPTI.

We next explored the robustness and evolvability of the BPTI sequence toward its main function, high-affinity binding to BT. We assumed that BPTI mutants that either stabilize the BPTI/BT complex or destabilize it by a small amount (1 kcal/mol or less) would be functional in the cellular environment. With this definition, only 2% of all single BPTI mutations were functional. The number of functional mutations was increased to ∼16% among the detected double mutations. However, among double mutations that involve the most energetically important position 15, only 5% would support high-affinity binding to BT. Both of the numbers for double mutations are likely an overestimation due to the absence of highly destabilized BPTI mutants from our data. We further compared the effect of the same BPTI mutations on its binding affinity to the three proteases by computing the correlation between ΔΔ*G*_bind_ values for the same mutations between pairs of PPIs. Our analysis shows that the same single mutations frequently result in similar binding affinity changes for all three PPIs (Figure S9A in the Supporting Information; *R* values of ∼0.55 for all PPI pairs), consistent with high sequence homology of the studied proteases. As expected, this correlation goes down to 0.3–0.4 when all single and double mutations are considered (Figure S9B–D in the Supporting Information). Further analysis shows that the largest majority of mutations in BPTI leads to a simultaneous decrease in affinity to pairs of proteases (91%, 81%, and 80% for BT/ChT, BT/MT, and ChT/MT pairs, respectively). Mutations that decrease affinity to the cognate protease BT but increase affinity to the homologous protease are observed with intermediate frequency (4.8% and 14.5% for BT/ChT and BT/MT, respectively). Mutations that increase affinity to the cognate protease BT but decrease affinity to the homologous protease are rather rare (2.9% and 2.7% for BT/ChT and BT/MT, respectively). Very rare yet detectable are BPTI mutations that simultaneously increase affinity for two proteases (1%, 1.6%, and 2% for BT/ChT, BT/MT, and ChT/MT, respectively). These results agree with previous studies on enzymes where the native activity was found to be less robust to mutations than the promiscuous activity.^62^ In addition, our results demonstrate the possibility of designing BPTI mutants that lead to an increase in binding specificity toward one particular protease.

Next, using the extensive quantitative data on ΔΔ*G*_bind_ for single and double mutants, we investigated the extent of coupling between various point mutations in BPTI when it interacts with the three proteases. We have classified mutations into three classes: additive and exhibiting positive and negative epistasis according to the magnitude of the coupling energy Δ*G*_*i*_ upon two mutations *X* and *Y*:
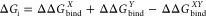
1Here, ΔΔ*G*_bind_^*X*^ and ΔΔ*G*_bind_^*Y*^ represent the change of the binding free energy of the single mutants *X* and *Y*, respectively, ΔΔ*G*_bind_^*XY*^ represents the change of binding free energy of the double mutant containing mutations *X* and *Y*. Negative epistasis was defined when Δ*G*_*i*_ < 0 within the uncertainties of the ΔΔ*G*_bind_ predictions for the double mutant and the two corresponding single mutants (eq 8), i.e., the double mutation exhibited lower affinity compared to what is expected from additivity of the two single mutations. Positive epistasis was defined when Δ*G*_*i*_ > 0, within the uncertainties of the ΔΔ*G*_bind_ predictions (eq 9), i.e., the double mutation exhibited higher affinity compared to what is expected from additivity of two single mutations. Two mutations were defined as additive if the mutations did not fall into either positive or negative epistasis groups, that is, Δ*G*_*i*_ = 0 within the uncertainties of the ΔΔ*G*_bind_ predictions. In such an analysis, we are likely overestimating the number of additive mutations as mutations with small epistasis and large uncertainties would be assigned into the “additive” group.

**Figure 7:**
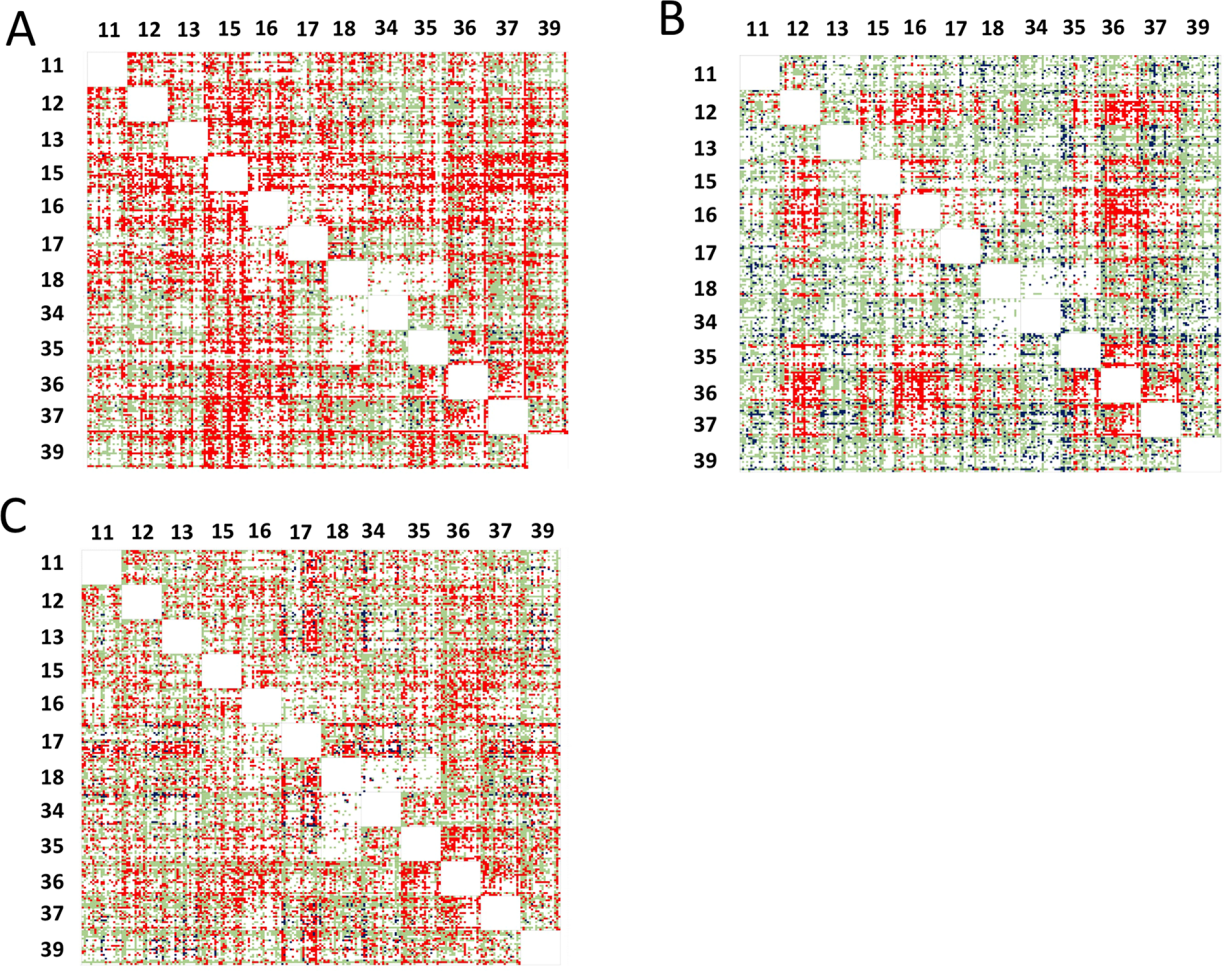
The matrix of ΔGi for double mutations in the three PPIs: (A) BT/BPTI; (B) ChT/BPTI; and (C) MT/BPTI. On the left and on the top are BPTI binding interface positions randomized to 20 amino acids. Each square is a 20 × 20 matrix containing ΔGi values for coupling between a particular mutation at one position to another particular mutation at another position in the same order. Color coding shows the degree of cooperativity. Green: additivity; red: positive epistasis; blue: negative epistasis; white: no data are available.

**Figure 8:**
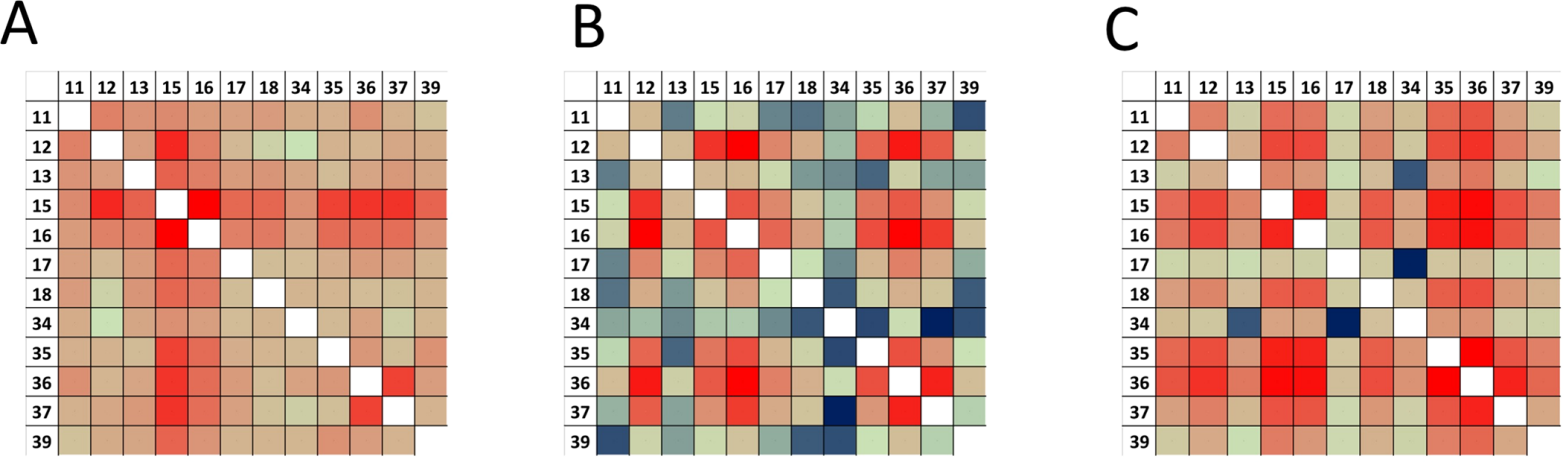
Coupling energies ΔGi averaged over all detected mutations at a pair of BPTI positions: (A) BT/BPTI interaction; (B) ChT/BPTI interaction; (C) MT/BPTI interaction. On the top and on the left BPTI positions are shown. The values are color coded from high positive epistasis (dark red) to additive (green) and to negative epistasis (dark blue).

Coupling energy analysis shows that in the BT/BPTI interaction 59% of the detected mutations are additive, 40% of mutations show positive epistasis, and only ∼1% of mutations show negative epistasis. In the ChT/BPTI interaction, 74% of mutations are additive, 18% of mutations show positive epistasis, and 8% of mutations show negative epistasis. Finally, among the detected mutations in the MT/BPTI interaction we observe 54% of mutations are additive, 42% exhibit positive epistasis, and 4% show negative epistasis. Note that among the invisible double mutations, the majority significantly destabilize BPTI folding. Such destabilizing mutations are likely to exhibit negative epistasis; thus, their absence from our data is consistent with a higher percentage of positive vs negative epistasis for all three complexes. We further constructed the per-position correlation matrices displaying coupling energy between all detected mutations in the three PPIs (Figure 7). Figure 7 shows that the sign of Δ*G*_*i*_ depends not only on a pair of positions but also on the mutation type, thus demonstrating multiple examples of sign epistasis.^63^ Yet, a certain preference for either negative or positive epistasis frequently dominates coupling at certain position pairs, as some squares are predominantly red or blue in Figure 7. To analyze which positions exhibit a higher degree of coupling, we averaged Δ*G_i_* values over all detected mutations at each pair of positions (Figure 8). Figure 8 shows that different proteases exhibit different patterns of coupling between pairs of mutations. Interestingly, positions where highest destabilization is observed for single mutations show a high degree of positive epistasis with all other positions (for example, positions 15 and 16 in the BPTI/BT complex, positions 12, 16, and 36 in the ChT/BPTI complex, and positions 15, 16, 35, and 36 in the MT/BPTI complex). On the other hand, cold-spot positions tend to exhibit negative epistasis with many other positions in the protein (see, for example, positions 13, 18, 34, and 39 in the ChT/BPTI complex and position 34 in the MT/BPTI complex). The average coupling energies are larger for the highest-affinity BT/BPTI complex, medium for the CT/BPTI complex, and the lowest for the MT/BPTI complex, in agreement with the overall dynamic range of ΔΔ*G*_bind_ values exhibited by the three complexes (Figure S10 in the Supporting Information). We further analyzed whether nonadditive mutations of the same ΔΔ*G*_bind_ sign exhibited antagonistic or synergetic epistasis; that is, the effect of combination of the two mutations resulted in smaller (antagonistic) or larger (synergetic) change compared to additivity. We found that antagonistic epistasis predominated in all three PPIs with 95%, 78%, and 99% of mutations in the BT/BPTI, ChT/BPTI, and MT/BPTI complexes, respectively. When combining a stabilizing mutation with a destabilizing mutation, affinity improvement was recorded for 92%, 55%, and 23% of nonadditive double mutations for the BT/BPTI, MT/BPTI, and ChT/BPTI complexes, respectively.

We further tested whether the degree of coupling between the two mutations depends on the distance between the mutated positions (Figure S11 in the Supporting Information). Our results show that mutations at two closely located positions could exhibit various degrees of coupling from high to low. As the distance between positions increases, the average coupling between the two mutations decreases (Figure S11 in the Supporting Information). A similar trend has been observed for all three PPIs and is in agreement with previous studies in various biological systems.^64^

## Discussion

### Link between PPI Binding Landscape and Its Function

**Figure 9:**
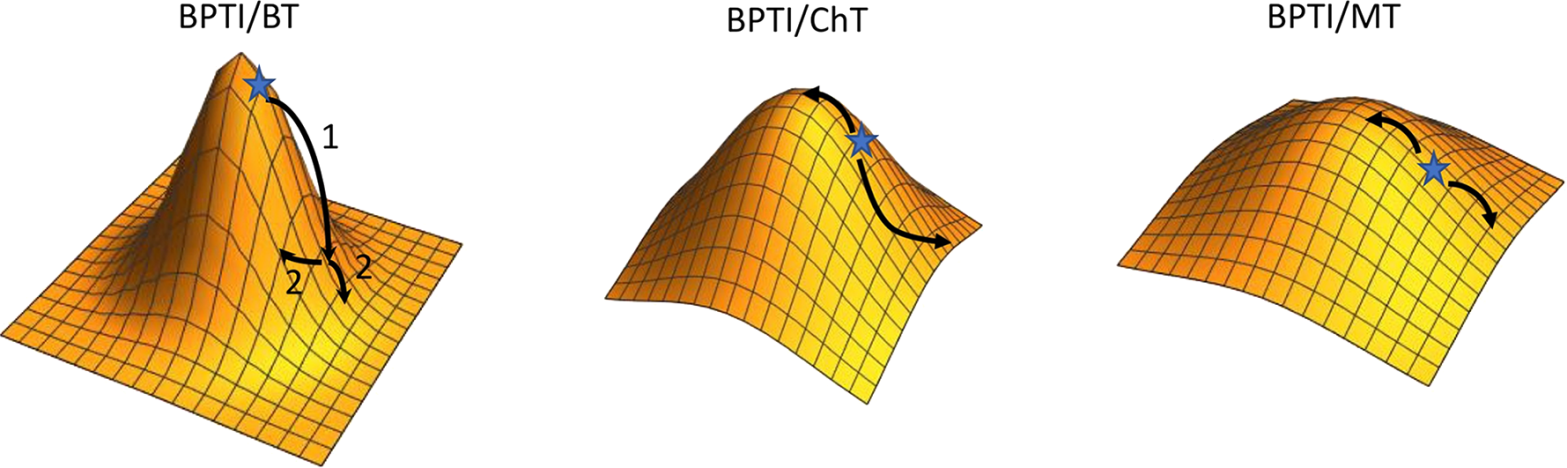
Schematic illustration of the single mutant binding landscapes for the three studied PPIs. The maximum on the surface corresponds to the highest possible binding affinity. A star indicates the position of the WT BPTI sequence with respect to the maximum. Arrows illustrate how single mutations lead to affinity changes in the three complexes.

In this study we measured quantitative effects of tens of thousands of single and double mutational steps in three homologous enzyme–inhibitor complexes. While the complexes are similar in their sequences and structures, they differ greatly in binding affinities that range from ultrahigh to low. We find that the binding landscape of each PPI depends strongly on the interaction *K*_D_. In particular, the ultrahigh affinity BT/BPTI complex is highly optimized in its sequence. Accordingly, the sequence of WT BPTI lies nearly at the maximum of the single mutant binding landscape (Figure 9), with only one mutation leading to significant affinity improvement. The landscape also exhibits a steep gradient, with a majority of single mutations leading to large steps down the hill with a maximum drop of ∼12 kcal/mol and an average drop of 4.5 kcal/mol (Figures 4A and 5A). Such high destabilizations from single mutations are extremely rare. For example, the SKEMPI database^65^ that reports 5079 single mutant binding affinity changes in various PPIs contains only 16 single mutations (0.3%) with ΔΔ*G*_bind_ values greater than 8 kcal/mol. Functionally, this result means that BPTI cannot accept mutations at key positions without losing its main function, i.e., high-affinity binding to BT. The medium-affinity ChT/BPTI complex shows a lower degree of optimality, with a larger fraction of single mutations leading to affinity improvement (16%) and a maximum improvement of 2.6 kcal/mol. Yet even in this complex, single mutational steps could lead to high complex destabilization of up to 6 kcal/mol. Thus, the single mutant landscape of the ChT/BPTI complex exhibits a medium gradient and the WT sequence lies about two-thirds (6 /(6 + 2.6)) up the landscape hill (Figure 9). One might expect that the low-affinity MT/BPTI complex would exhibit a ΔΔ*G*_bind_ distribution that is the opposite of that observed for the BPTI/BT complex, with a high number of mutations that lead to a very large improvement in binding affinity. Yet, this is not what we observed in the present study. The MT/BPTI complex indeed exhibits the highest fraction of mutations leading to affinity improvement among the three complexes (22%), but the largest improvement due to a single mutation does not exceed 1.9 kcal/mol, smaller than what is observed for the ChT/BPTI complex. Yet, the reduction in binding affinity due to single mutations is also the smallest for the MT/BPTI complex, not exceeding 3 kcal/mol. Thus, we conclude that the difference between the MT/BPTI and BT/BPTI complexes is not only the location of their sequences relative to the maximum of the binding landscape but the single mutant landscapes themselves show different gradients, high for the high-affinity complex and low for the low-affinity complex (Figure 9).

The binding landscape characteristics of the three studied PPIs have been dictated by their evolutionary history. BPTI has coevolved with BT to optimize their affinity for each other, as a mechanism to protect the pancreas. Activation of trypsin is normally catalyzed by enteropeptidase in the duodenum and serves as a the master regulatory step in digestive enzyme activation, since trypsin is the common activator of all other pancreatic zymogens.^66^ Premature autoactivation of trypsin in the pancreas can cause a runaway activation cascade, leading to tissue damage, inflammation, and pancreatitis.^66^ Thus, BPTI and other potent pancreatic trypsin inhibitors have evolved to regulate this key digestive enzyme. Humans do not possess an exact ortholog of BPTI, but the same biological protective function is filled by SPINK1,^66^ another canonical trypsin inhibitor that has coevolved with human trypsin.

By contrast, ChT and MT are not susceptible to autoactivation and lack specific endogenous coevolved inhibitors in the mammalian pancreas. They bind to BPTI due to their sequence and structural homology to BT and due to the preconfiguration of the BPTI binding loop in canonical conformation with complementarity to conserved features of the serine protease active site.^51,52^ These nonoptimized PPIs notably show weaker affinity than that exhibited by BPTI/BT. The interaction with MT is particularly weak, despite the similar specificity of BT and MT for cleavage after Lys/Arg, due to the evolution of MT for widespread natural resistance to the canonical trypsin inhibitors such as BPTI.^67,68^ Furthermore, by binding to these inhibitors orders of magnitude more weakly than other trypsins, MT has evolved the capability to cleave many proteinaceous trypsin inhibitors as substrates.^54,56,69−71^ The influence of this evolutionary adaption on the MT/BPTI interaction is consistent with our finding that no single or double mutation on the BPTI side could convert this complex into a high-affinity PPI. We anticipate that BPTI may yet be converted to a high-affinity inhibitor of MT but in this case requiring a greater number of combined mutations. Consistent with such a possibility, APPI, a human BPTI paralog with 44% sequence identity, binds to MT ∼100-fold more tightly (*K*_D_ ≈ 10^–7^ M),^50^ while triple and quadruple mutants of APPI identified by directed evolution can bind to MT more than 1000-fold yet more tightly (*K*_D_ < 10^–10^ M).^72,73^

### Comparison of Cold-Spot and Hot-Spot Locations in the Three PPIs

Cold-spots are positions in proteins that are occupied by nonoptimal amino acids; at such positions, multiple mutations lead to functional improvement. In this study we analyzed cold-spot and hot-spot locations on BPTI and found several notable differences between the three studied PPIs. The most prominent difference is observed at position 15, which is nearly a hot-spot in the BT/BPTI and the MT/MPTI complexes but is a cold-spot in the ChT/BPTI complex. This central to the binding interface position is occupied by Lys in WT BPTI, which fits perfectly in the BT specificity pocket, where it participates in a network of hydrogen bond interactions with several polar residues on BT (Figure S12 in the Supporting Information). The K15 binding pocket is completely conserved in the MT/BPTI complex. In agreement with structural identity of the MT binding pocket, all mutations but mutation to Arg at position 15 result in destabilization of the MT/BPTI complex, yet mutational changes are smaller in comparison to those exhibited by the BT/BPTI interaction. On the contrary, in the ChT/BPTI interaction, mutations to all hydrophobic amino acids at position 15 lead to improved affinity. Here, slight differences in the ChT specificity pocket prevent the formation of the hydrogen bond network with K15 of BPTI; hence K15 exhibits a different, less buried conformation, avoiding unfavorable charge burial (Figure S12 in the Supporting Information).

Additional differences in cold-spot and hot-spot locations are observed at the periphery of the BPTI/protease binding interface, where they could be explained from the structural perspective. Affinity-enhancing mutations at cold-spots could occur either through removal of an unfavorable interaction with the partner protein or through introduction of a new favorable interaction, where no interaction exists.^49^ We observed the first scenario occurring in the MT/BPTI complex at position 17, where an Arg on BPTI is found in close proximity to Arg 193 on MT, resulting in unfavorable charge repulsion (Figure S13 in the Supporting Information). As our experimental data show, substituting Arg 17 with a small and/or hydrophobic amino acid results in affinity improvement (Figure 4C). On the contrary, Arg 17 is found in a largely hydrophobic environment in the ChT/BPTI complex; its replacement with a hydrophobic Met and Leu results in slightly negative ΔΔ*G*_bind_ values (Figure 4B). A similar scenario is observed at position 39, which is a cold-spot in the MT/BPTI and ChT/BPTI complexes but a hot-spot in the BT/BPTI complex. Here, Arg 39 on WT BPTI is found in close proximity to Lys 175 on MT and ChT; Arg 39 substitution by a noncharged residue, such as Trp, results in affinity improvement in both complexes (Figure S14 in the Supporting Information). In the BT/BPTI complex, position 175 is occupied by Gln, which forms a hydrogen bond with Arg 39 on BPTI, explaining the hot-spot nature of this position in the BT/BPTI complex.

We observe the second scenario for cold-spot formation at position 34 in the ChT/BPTI and MT/BPTI complexes. Val 34 at this position does not form any interactions with these proteases. Its replacement with larger hydrophobic amino acids that bury additional surface area increases affinity to ChT. Its replacement with polar or negatively charged residues improves affinity to MT by likely forming new hydrogen bonds to Tyr 151 and/or Gln 192 on MT.

### Epistasis in Protease/BPTI Complexes

Using the data for tens of thousands of double mutants we were able to analyze how two mutations are coupled in the three protease/BPTI complexes. Our data show that in all three BT/BPTI complexes a large proportion of double mutations results in positive epistasis and only a minority of mutations produces negative epistasis. We also observe the predominance of antagonistic epistasis vs synergetic epistasis. This absence of symmetry is at least partially due to the absence of highly destabilizing double mutations from our data. The abundance of positive epistasis in the BT/BPTI interaction could be explained from the perspective of binding landscape theory (Figure 9). Due to the steepness of the gradient in the area of the wild-type BPTI sequence, the first mutation in this PPI leads to a large step down the hill into the area of low gradient. A second mutation from this point could lead up or down, but the change would be relatively small, resulting in positive epistasis, i.e., better ΔΔ*G*_bind_ compared to what would be predicted from additivity of the two highly destabilizing mutations. Positive epistasis particularly predominates at positions where the largest affinity drops are recorded (such as at positions 15 and 16 for the BT/BPTI complex or position 12 for the ChT/BPTI complex), where the gradient is steep.

Positive epistasis could be also explained from the structural perspective. Highly optimized PPIs usually retain their original binding conformation upon introduction of a single mutation due to the abundance of favorable interactions generated at nonmutated positions. Yet, if any of the hot-spot residues is mutated (such as K15 in the BPTI/BT and BPTI/MT complex), substantially weakening the interaction, then the impact of a second deleterious mutation may be mitigated by an increase in flexibility at the interface, enabling the protein to adopt alternative conformations that introduce new favorable intermolecular contacts and enhance affinity. If the same mutation would occur on the background of the wild-type residue in the hot-spot position, the new conformation would not be accessible and the affinity enhancement would not be achieved. Thus, such a double mutant would possess better ΔΔ*G*_bind_ compared to the sum of single mutants, exhibiting positive epistasis.

On the contrary, negative epistasis is more frequent for medium- and low-affinity PPIs and appears mostly when one mutation is performed at a cold-spot position. If the first mutation improves binding affinity and thus makes a step up the binding landscape toward the maximum, the second mutation would be made from the point of steeper gradient and is likely to make a large step down, thus resulting in negative epistasis. Structurally that means that if at one cold-spot position a new favorable interaction was created, this interaction might lock the PPI into a new slightly different conformation. Another conformation might be acquired upon introduction of a different affinity-enhancing mutation. But the two favorable conformations could not be achieved simultaneously, resulting in worse ΔΔ*G*_bind_ for a double mutation compared to the sum of two single mutations (negative or antagonistic epistasis).

In summary, in this study we report ΔΔ*G*_bind_ values for tens of thousands of single and double mutations in three protease/BPTI complexes with similar structures but highly variable binding affinities, thus generating an unprecedented amount of mutational data that could be used as a benchmark for testing new computational methodology and for the design of new high-affinity protease inhibitors. Using the obtained data, we demonstrate striking differences between the single mutant binding landscapes of the three PPIs that could be explained by the level of the PPI evolutionary optimality. Furthermore, we study how two single mutations in these PPIs couple to each other and demonstrate that the coupling energy depends not only on positions of mutations but also on the identities of the mutated amino acid. Furthermore, we observe that mutations at hot-spot positions generally exhibit positive epistasis with other mutations, while mutations at cold-spot positions generally exhibit negative epistasis and explain this phenomenon from the perspective of binding landscape theory. Our powerful experimental methodology could be used to access the binding landscapes in many additional PPIs with different structures, functions, and affinities and to probe whether the reported evolutionary trends hold in other biological systems.

## Methods

### BPTI Library Construction

Twelve positions on BPTI that lie in the binding interface with BT (PDB ID 3OTJ) were subject to randomization: T11, G12, P13, K15, A16, R17, I18, V34, Y35, G36, G37, and R39. A BPTI library was constructed that randomized two positions at a time with an NNS codon (where N = A/C/G/T DNA base, S = C/G DNA base), encoding all amino acids at the randomized positions, including the WT amino acid. The library was divided into 66 sublibraries that each incorporates all possible pairs of the 12 randomized positions. The TPCR protocol^74^ was used to create each library using two primers that either combined two mutations in one primer or divided them into two primers depending on their proximity to each other (Supplementary Note 1 in the Supporting Information). These primers were used in a PCR together with the BPTI_WT_ plasmid to incorporate these mutations at the specific positions in BPTI and to amplify the whole plasmid. Agarose gel analysis was used to confirm the success of each TPCR reaction. The TPCR-amplified plasmid DNA was treated with DpnI (New England Biolabs, Ipswich, MA, USA) to remove any parental plasmid used as a template to construct the library, cleaned up with magnetic beads (AMPure XP, Beckman Coulter, Brea, CA, USA), and transferred into *E. coli*, and selected colonies were sequenced to confirm the successful generation and transformation of the BPTI library. The vectors containing the BPTI library were extracted using QIAprep Spin miniprep (Qiagen, Hilden, Germany), and all the sublibraries were pooled together and balanced by their DNA amount to use the same amount of DNA from each sublibrary (∼3.6 μg). Then, the pooled library was transferred into *S. cerevisiae* using 20 transformations, resulting in 60 000–70 000 colonies for the complete library as estimated by plating 1/20 the amount of the library sample and counting the colonies after transformation on a SDCAA plate.

### YSD Sorting Experiments

Yeast cells displaying the BPTI library or the BPTI_WT_ with a cMyc-tag at the C-terminus on the YSD were grown in SDCAA selective medium and induced for BPTI protein expression with a galactose-containing SGCAA medium as previously described.^72^ BPTI expression and binding to individual proteases were detected by incubating approximately 1 × 10^6^ yeast cells with a 1:50 dilution of mouse anti-cMyc antibody (9E10, Abcam, Cambridge, UK) in 1× phosphate-buffered saline (PBS) supplemented with 1% bovine serum albumin (BSA, Thermo Fisher Scientific, Waltham, MA, USA) for 1 h at room temperature, washed with ice-cold 1× PBS, and then incubated with different concentrations of biotinylated BT (biotin and biotinylation protocol from Thermo Fisher Scientific) in 1× PBS with 1% BSA for 1 h at room temperature. Thereafter, cells were washed with ice-cold 1× PBS, followed by incubation with a 1:50 dilution of phycoerythrin (PE)-conjugated anti-mouse secondary antibody (Sigma-Aldrich, St. Louis, MO, USA) and NeutrAvidin (Thermo Fisher Scientific) conjugated with FITC in 1× PBS with 1% BSA for 20 min on ice. Finally, the cells were washed with ice-cold PBS, and the fluorescence intensity was analyzed by dual-color flow cytometry (Accuri C6, BD Biosciences). The yeast cells were next sorted into four populations by FACSAria (BD Biosciences, San Jose, CA, USA) including HI, WT, SL, and LO populations. Sorted cells were then grown in a selective medium, and the plasmidic DNA was extracted for each of the sorted population and the naïve library and submitted to NGS by MiSeq, Illumina (service provided by Hylabs, Rechovot, IL, USA).

### NGS Analysis

The paired-end reads from the NGS experiments were merged,^75^ and their quality scores were calculated in the FastQC tool (https://www.bioinformatics.babraham.ac.uk/projects/fastqc/). In the Matlab script, the sequences were aligned, and sequences containing extra mutations at nonrandomized positions were filtered out. The number of each remaining BPTI mutant *i* was counted in the sorted and the naïve populations, and its frequency *f*^*i*^ in the libraries was calculated (eq 2). Using the frequency of the mutant in one of the sorted populations and the naïve population, the enrichment *e*^*i*^ of each BPTI mutant was calculated (eq 3).
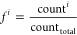
2
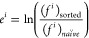
3To estimate the uncertainty in BPTI mutant frequencies, we applied a bootstrapping method to the NGS data for all sorted gates and the naïve library as described in ref (76). Briefly, the original NGS data were used to randomly draw sequences to obtain a resampling data set of the same size and to calculate the frequency of each BPTI mutant in each population. The resampling process was repeated 1000 times, and the average frequency and the standard deviation were calculated from 1000 resampling data sets for each BPTI mutant in each sorting gate and in the naïve library. The error was propagated into eqs 2 and 3 to calculate the error in enrichment values:
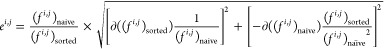
4The error values in enrichment values were propagated to calculate the errors in ΔΔ*G*_bind_ predictions.

### Predicting ΔΔ*G*_bind_ Values from NGS Data

All available experimental data on ΔΔ*G*_bind_ for the BPTI/protease complexes were used to obtain the best normalization formulas for each complex for converting enrichment values from the four sorted populations into ΔΔ*G*_bind_ values. To this end, we used a linear regression model function in Mathematica (Wolfram Research) with five parameters (*Y* = *aX*_1_ + *bX*_2_ + *cX*_3_ + *dX*_4_ + *f*) if all four enrichment values were available in our NGS data for this particular mutation. The parameters *a*, *b*, *c*, *d*, and *f* were optimized using the experimental data set as values of *Y* and the set of *X*_1_, *X*_2_, *X*_3_, and *X*_4_ enrichment values. The obtained normalization formula (different for each protease) was used to calculate ΔΔ*G*_bind_ values for all the remaining single and double BPTI mutants that had four enrichment values recorded in the NGS experiment.

The standard deviation of ΔΔ*G*_bind_ predictions for each BPTI mutant was calculated according to the formula
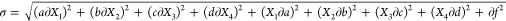
5Where a, b, c, d are the coefficients in front of *X*_1_, *X*_2_, *X*_3_, and *X*_4_, respectively; *∂X*_1_, *∂X*_2_, *∂X*_3_, and ∂X_4_ are the standard deviations on these variables obtained from the bootstrapping analysis of the NGS data, and *∂a*, *∂b*, *∂c*, *∂d*, and *∂f* are the standard deviations of these coefficients obtained from the leave-one-out analysis. To make ΔΔ*G*_bind_ prediction for mutants where fewer than four enrichment values were available, we repeated the normalization procedure using different subsets of enrichment values (such as *X*_1_ and *X*_4_; X_1_, *X*_2_, *X*_3_; etc.). Accordingly, we varied the number of parameters in the normalization formula. We checked whether high correlation with the experimental data set of ΔΔ*G*_bind_ values could be obtained using this particular subset of variables as predictors. If a correlation of *R* > 0.80 was obtained between the predicted and the experimental ΔΔ*G*_bind_ values, the set of gates was selected as good for making predictions. Additional cross check for validity of predictions from this subset of gates was performed by comparing ΔΔ*G*_bind_ predictions for all single mutants based on all four gates and based on the selected subset of gates and confirming high correlation between the two predicted ΔΔ*G*_bind_ values over all single mutations. For each of the mutants, we used the available enrichment values to make separate predictions from all possible “good” subsets of gates. First all predictions were recorded for mutants where enrichment values were available for all four gates. For mutants where predictions were available for only gates *X*_1_, *X*_2_, and *X*_4_, predictions were made based on these three gates providing that this set of gates was defined as good. For mutants where predictions were available in only gates *X*_1_ and *X*_4_ predictions were made based on these two gates if this set of gates was defined as good for predictions. For each prediction from each subset of gates, the uncertainty of the prediction was calculated by propagating an error from the enrichment values (see ref (57) for details). Finally, for each mutation, ΔΔ*G*_bind_ prediction was selected from all the predictions according to the subset of gates where the highest correlation with experimental data was observed. The data set of final ΔΔ*G*_bind_ predictions for all single and double mutants for the three PPIs could be found in the Source Data file.

### Production of BPTI Variants

The BPTI_WT_ sequence was cloned into a pPIC9K vector (Invitrogen, Carlsbad, CA, USA), and TPCR was used for site-directed mutagenesis to create the sequences of all variants, transformed, expressed in *P. pastoris* (*GS115* strain; obtained from Invitrogen) and purified by nickel affinity chromatography, followed by size-exclusion chromatography, as described in a previous work.^72^ The correct DNA sequence of each produced protein was confirmed by extracting the plasmidic DNA from *P. pastoris* after protein purification by nickel chromatography, amplifying the BPTI gene and sequencing it. Protein purity was validated by SDS-PAGE on a 20% polyacrylamide gel, and the mass was determined with a MALDI-TOF REFLEX-IV (Bruker) mass spectrometer (IKI, BGU; data not shown). Purification yields for all BPTI variants were 2–15 mg per liter of medium. The concentration of purified BPTI variants was determined by an activity assay.

For MT, values of the inhibition constant (*K*_i_) were determined using a general enzyme activity essay for PPIs characterized by medium to low affinity.^72^ Here, 304 μL of BPTI (four different concentrations ranging from 5.2 to 52.6 μM) was mixed with 8 μL of the substrate Z-GPR-pNA (Sigma-Aldrich) (five different concentrations ranging from 0.4 to 10 mM). The mix was incubated for 3 min. Then, the reaction was initialized by adding 8 μL of MT (10 nM) and the absorbance of the samples was measured at a wavelength of 410 nm for 5 min. A negative control was added replacing BPTI with 304 μL of buffer (10 mM Tris, pH 8, 1 mM CaCl_2_). The range of concentrations of BPTI was adapted when the determined *K*_i_ was not in the range of these BPTI concentrations.

For MT, the *K*_i_ could be determined from eq 6, as described previously.^72^
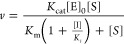
6The change in experimental binding energy ΔΔ*G*_bind_ was calculated from eq 7 using the *K*_i_ of the WT and the mutant, the temperature *T* at which the affinity was measured, and the ideal gas constant *R*.
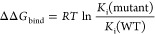
7

### Analysis of Additivity and Cooperativity

For each double mutation with available ΔΔ*G*_bind_ prediction, we calculated the interaction energy between the two single mutations according to eq 1.

The mutation was defined as exhibiting negative epistasis if Δ*G*_i_ was negative within the uncertainty of the predictions, that is,
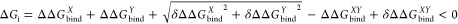
8Here, δΔΔ*G*_bind_^*X*^, δΔΔ*G*_bind_^*Y*^, and δΔΔ*G*_bind_^*XY*^ are uncertainties in prediction of ΔΔ*G*_bind_ for mutation *X*, *Y*, and *XY*, respectively.

The mutation was defined as exhibiting positive epistasis if Δ*G*_i_ was positive within the uncertainty of the predictions, that is,
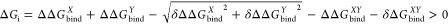
9If the value of Δ*G*_i_ fell between the values of positive or negative epistasis, the mutation was defined as additive.

## Supporting Information Available

Source Data file: data for all ΔΔ*G*_bind_ predictions for single and double BPTI mutants interacting with the three proteases (XLSX)
FACS data for experimental optimization and YSD selection experiments, MT/BPTI mutant binding affinity data, NGS normalization data for all PPIs, analysis of the binding landscapes for some single BPTI mutants, pairwise correlations between ΔΔ*G*_bind_ values, absolute values of coupling energies, dependence of coupling on the Ca–Ca distance, structural modeling figures (PDF)
