# Supplementary material for: Climbing Up and Down Binding Landscapes through Deep Mutational Scanning of Three Homologous Protein–Protein Complexes
Source: J Am Chem Soc. 2021 Oct 5;143(41):17261–75. doi: 10.1021/jacs.1c08707 (PMC8532158; doi:10.1021/jacs.1c08707)
Supplement: Supplementary file 2 — ja1c08707_si_002.pdf [file ja1c08707_si_002.pdf]

Supplementary Information for

Climbing up and down binding landscapes through deep mutational scanning of three homologous protein-protein complexes

Michael Heyne[1,2], Jason Shirian[1], Itay Cohen[2], Yoav Peleg[3], Evette S. Radisky[4], Niv Papo[2*], and Julia M. Shifman[1*]

[1]Department of Biological Chemistry, The Alexander Silberman Institute of Life Sciences, The Hebrew University of Jerusalem, Jerusalem, 9190401, Israel

[2]Avram and Stella Goldstein-Goren Department of Biotechnology Engineering and the National Institute of Biotechnology in the Negev, Ben-Gurion University of the Negev, Beer-Sheva, 8410501, Israel

[3]Life Sciences Core Facilities (LSCF) Structural Proteomics Unit (SPU), Weizmann Institute of Science, Rehovot, 7610001, Israel

[4]Department of Cancer Biology, Mayo Clinic Comprehensive Cancer Center, Jacksonville, Florida, 32224, USA

* To whom correspondence should be addressed. E-mail: jshifman@mail.huji.ac.il; papo@bgu.ac.il

This PDF file includes:

FACS data for $BPTI_{WT}$ bound to 3 serine proteases: **Figure S1**
FACS data for optimizing protease concentration for YSD: **Figure S2**
FACS data for YSD selections into 4 affinity windows for the 3 proteases: **Figures S3-S5**
Kd measurements for the twelve BPTI mutants binding to MT: **Figure S6**
Correlation between $\Delta\Delta G_{bind}$ predicted from NGS and experimental $\Delta\Delta G_{bind}$ measured on purified proteins: **Figure S7**
Histograms of $\Delta\Delta G_{bind}$ values due to single mutational steps for BT interacting with 3 BPTI single mutants: **Figure S8**
Pairwise correlations between $\Delta\Delta G_{bind}$ values for the same mutations in different PPIs: **Figure S9**
The absolute values of coupling energies ΔGi averaged over a pair of BPTI positions: **Figure S10**
Dependence of ΔGi on the Ca-Ca distance between the two positions: **Figure S11**
Structural comparison of the K15 binding site in the 3 proteases: **Figure S12**
Structural comparison of the R17 binding site in the 3 proteases: **Figure S13**
Structural comparison of the R39 binding site in the 3 proteases: **Figure S14**
Primers for construction of BPTI library: **Supplementary Note 1**

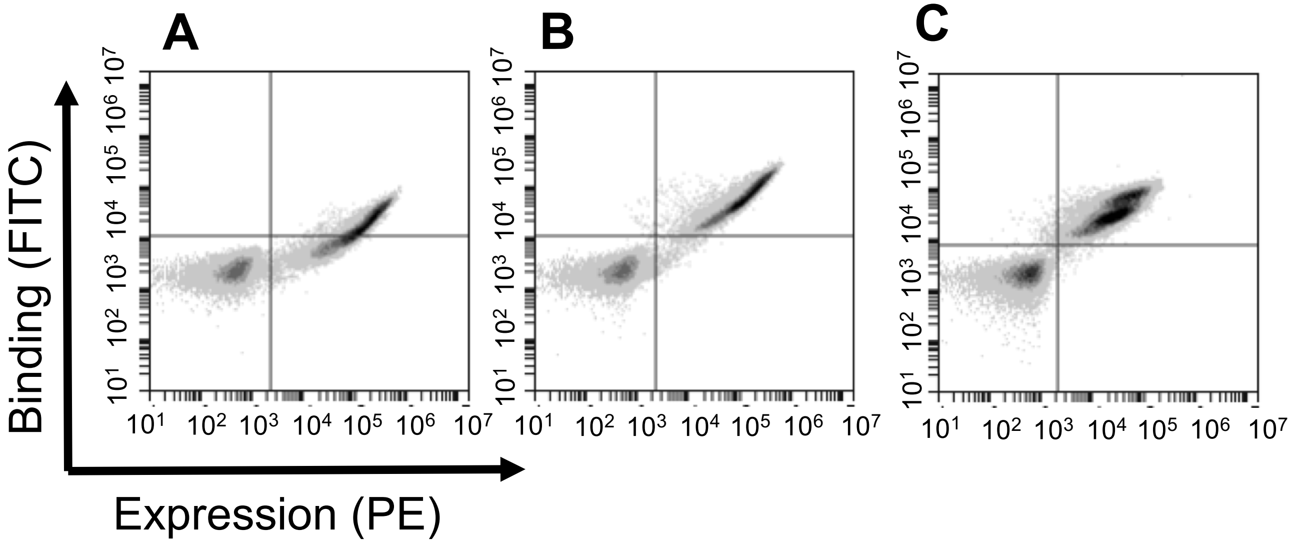

Figure S1: FACS data of $BPTI_{WT}$ binding to A) 100 nM MT, B) 20 nM ChT, C) 5 nM BT. Expression of $BPTI_{WT}$ was monitored via fluorescence of PE while binding to the three proteases was monitored via FITC fluorophore conjugated to the trypsin.

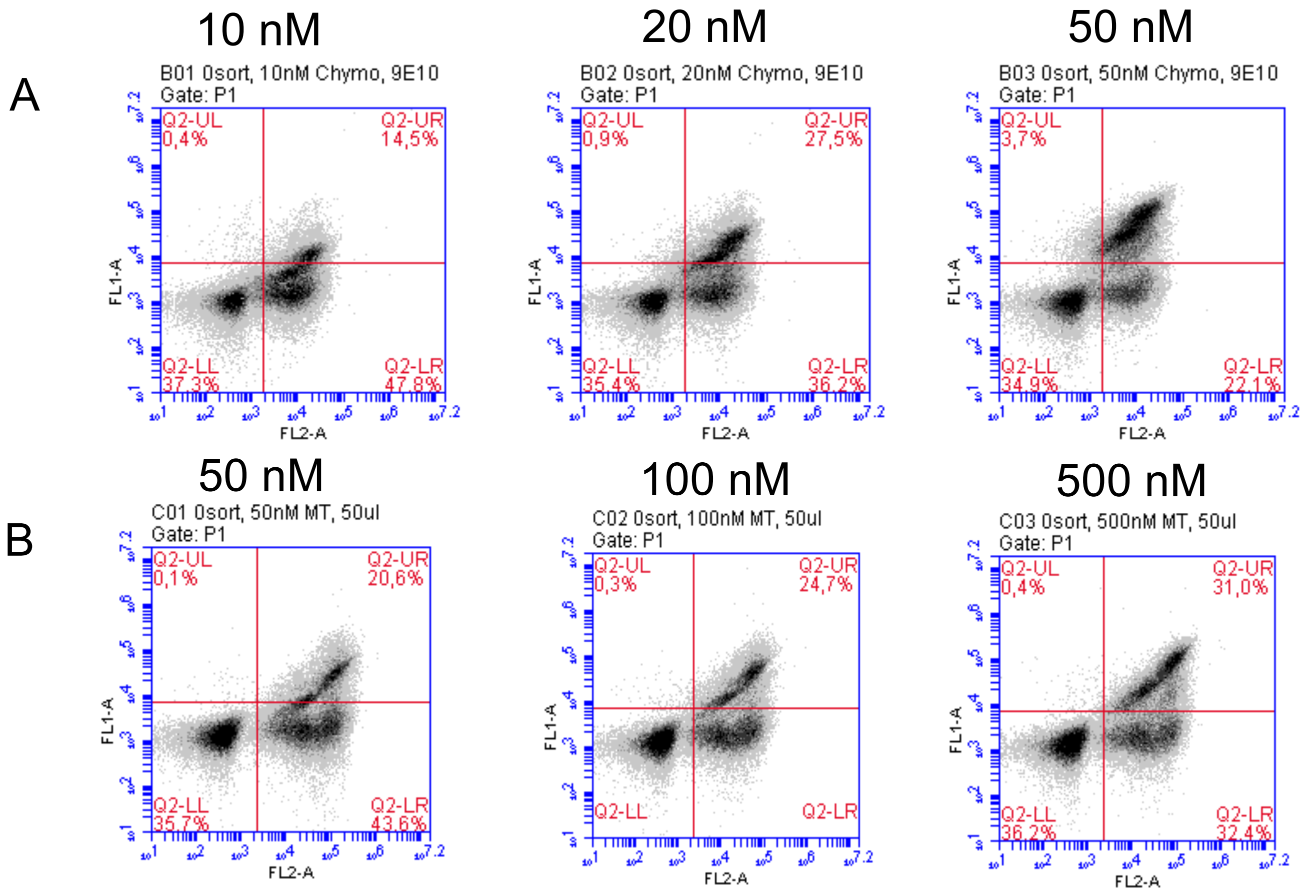

Figure S2: Optimizing protease concentration for YSD selection. Various concentrations of ChT (A) and MT (B) were incubated with the BPTI library and the FACS signal was measured. The concentration for BT was optimized in our previous work to be 5 nM (ref. 57). Concentration for sorting was set to 20 nM for ChT and 100 nM for MT.

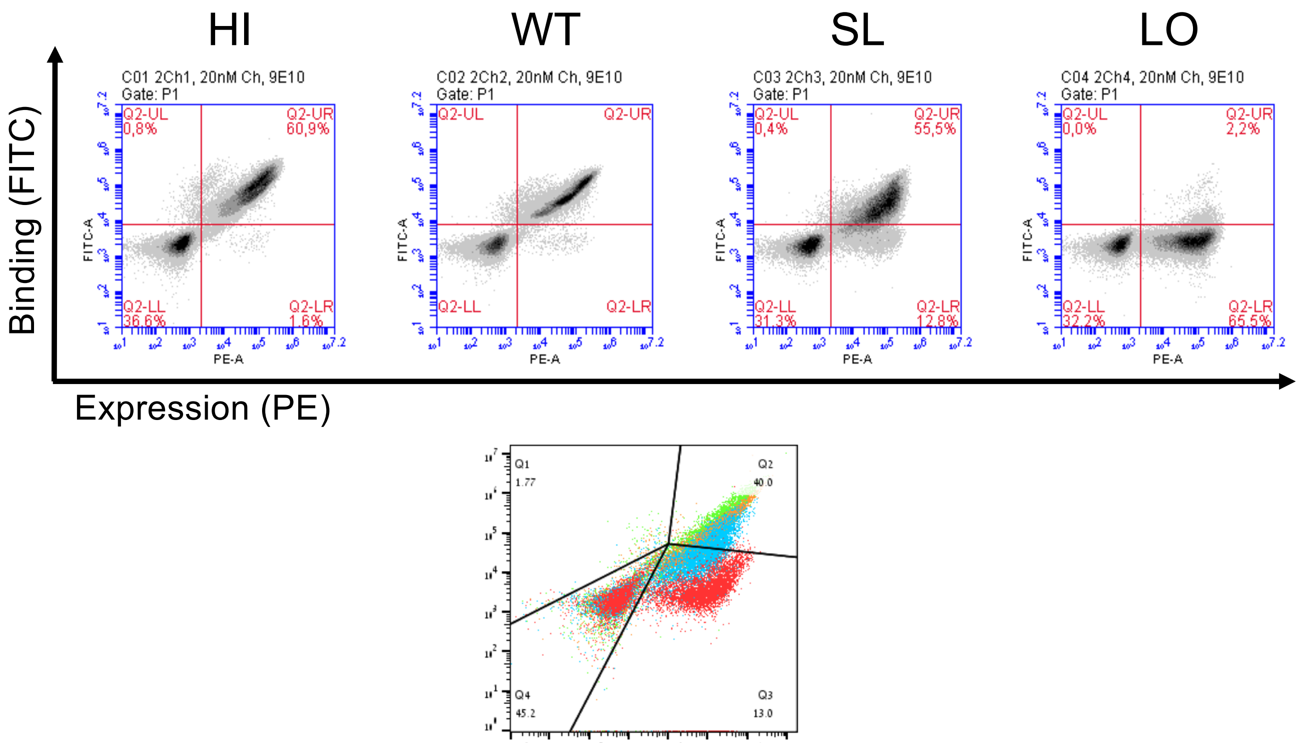

Figure S3 Upper panel: Separation of BPTI mutant clones into four affinity windows when binding to ChT. The FACS analysis after yeast cells were sorted into 4 gates and each sorted population of cells was re-grown. Lower panel: superposition of the four populations showing cells from the LO gate in red, from SL gate in blue, from WT gate in orange and HI gate in green.

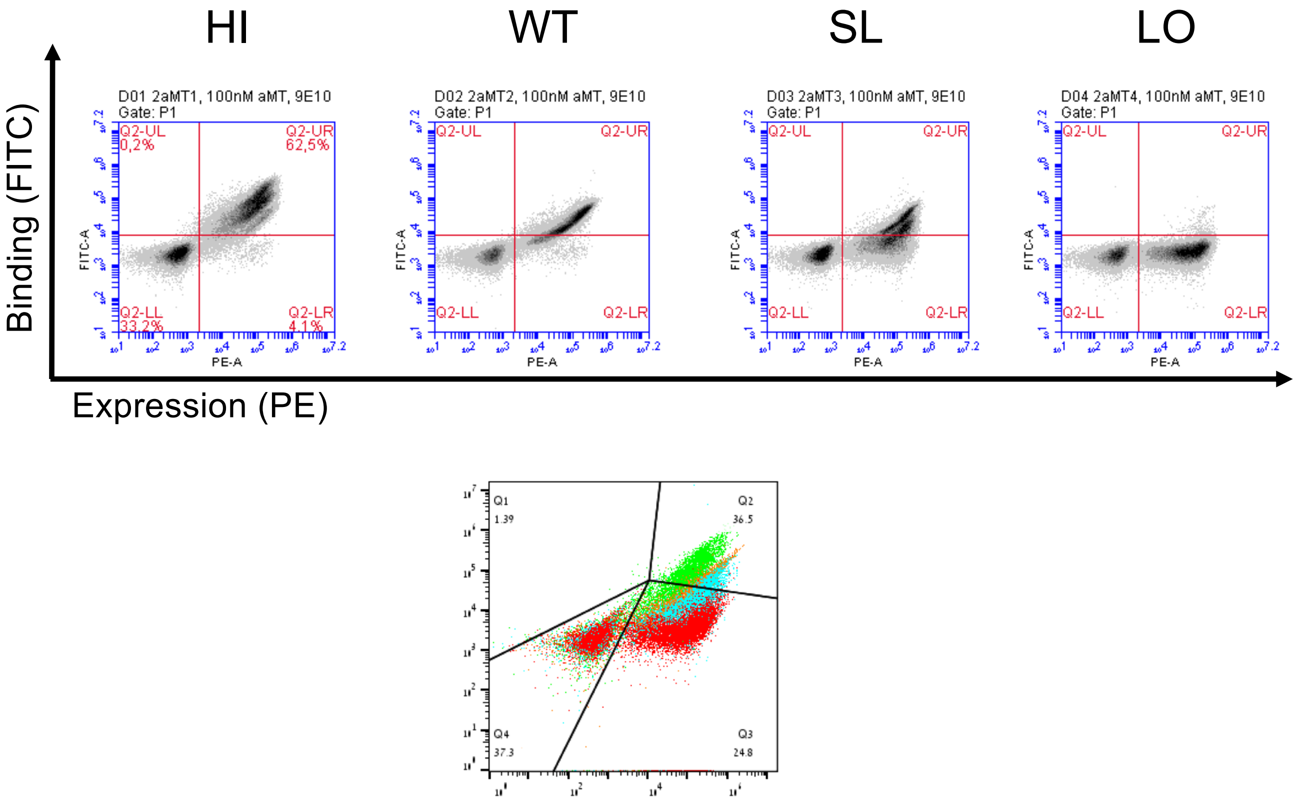

Figure S4 Upper panel: Separation of BPTI mutant clones into four affinity windows when binding to MT. The FACS analysis after yeast cells were sorted into 4 gates and each sorted population of cells was re-grown. Lower panel: superposition of the four populations showing cells from the LO gate in red, from SL gate in blue, from WT gate in orange and HI gate in green.

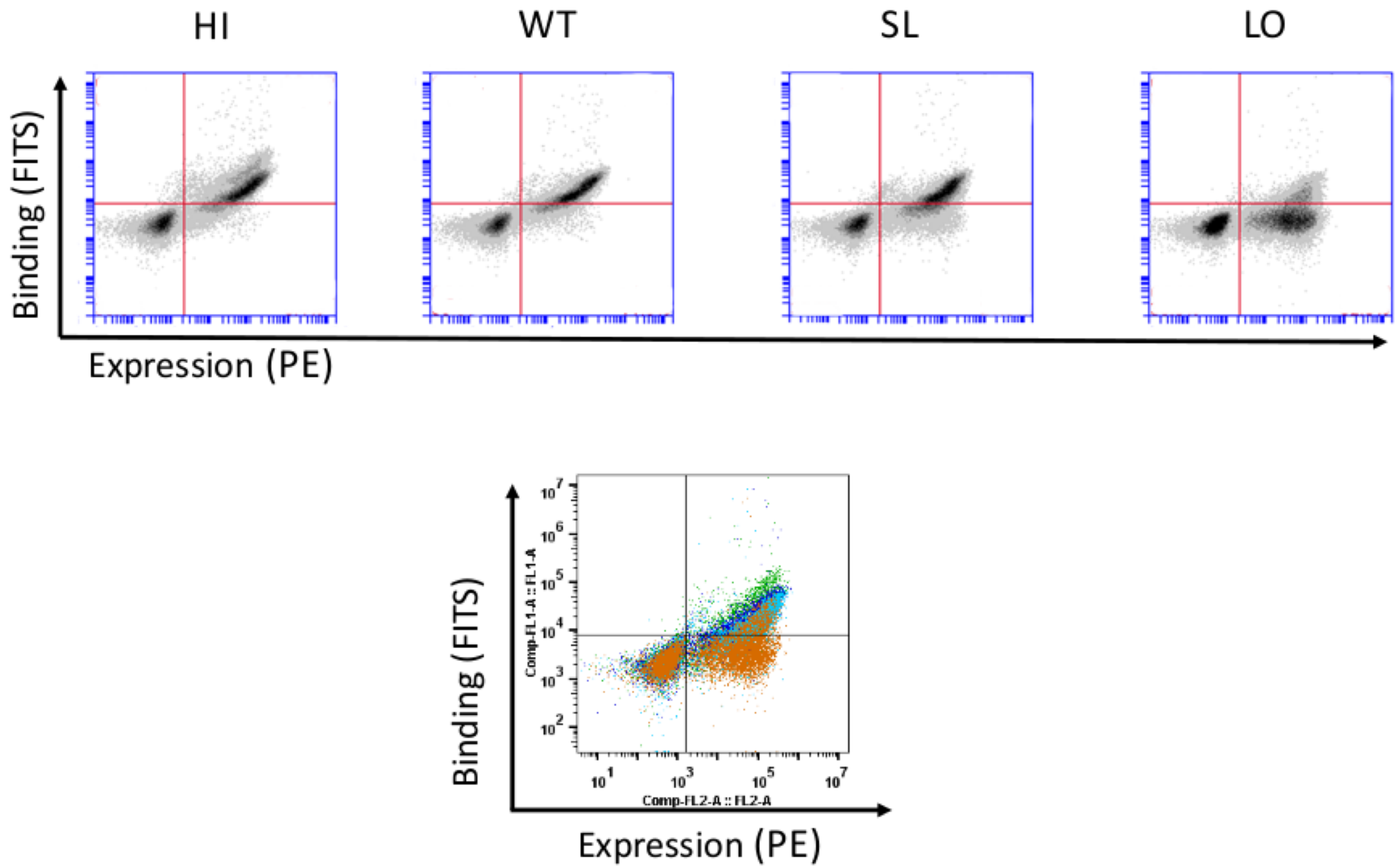

Figure S5 Upper panel: Separation of BPTI mutant clones into four affinity windows when binding to BT. The FACS analysis after yeast cells were sorted into 4 gates and each sorted population of cells was re-grown. Lower panel: superposition of the four populations showing cells from the LO gate in orange, from SL gate in light blue, from WT gate in dark blue and HI gate in green.

(A)

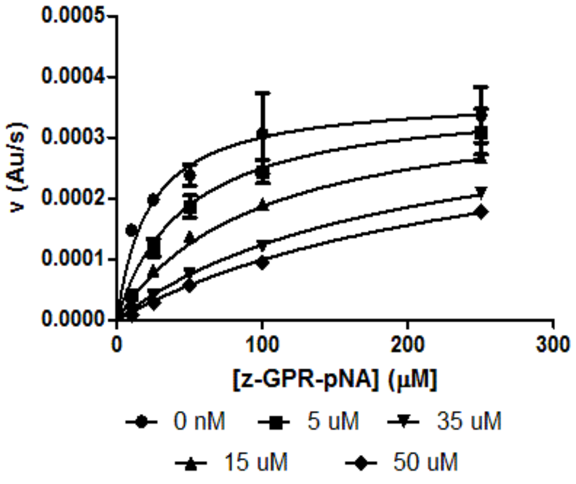

(B)

Mutant	K_i ± S. D. (μM)	$\Delta\Delta G_{exp}$ (kcal/mol)
WT	4.49 ± 0.57	0.00 ± 0.13
T11N	32.02 ± 5.12	1.21 ± 0.09
P13L	6.46 ± 0.52	0.22 ± 0.05
P13Y	2.33 ± 0.28	-0.40 ± 0.07
K15R	1.14 ± 0.17	-0.81 ± 0.09
R17L	0.79 ± 0.04	-1.07 ± 0.03
I18K	12.51 ± 1.97	0.63 ± 0.09
V34D	0.22 + 0.03	-1.86 ± 0.08
V34H	6.97 + 0.68	0.27 ± 0.06
V34W	9.26 + 0.90	0.45 ± 0.06
R39I	1.50 + 0.15	-0.68 ± 0.06
T11N+V34H	13.48 ± 2.07	0.68 ± 0.09

Figure S6: Measurement of $\Delta\Delta G_{bind}$ values for purified MT binding to BPTI mutants
(A) Representative experiment for determination of Ki values of BPTI mutants for MT. $BPTI_{WT}$ was used in this experiment and similar experiments were repeated with all BPTI mutants. MT cleavage of the peptide substrate Z-GPR-pNA was measured in the absence and in the presence of inhibitor, $BPTI_{WT}$. The experiment was repeated using several concentrations of $BPTI_{WT}$ as shown at the bottom. (B) Experimental results for the binding energy between MT and the BPTI variants determined in this study and used for normalization of enrichment values for MT.

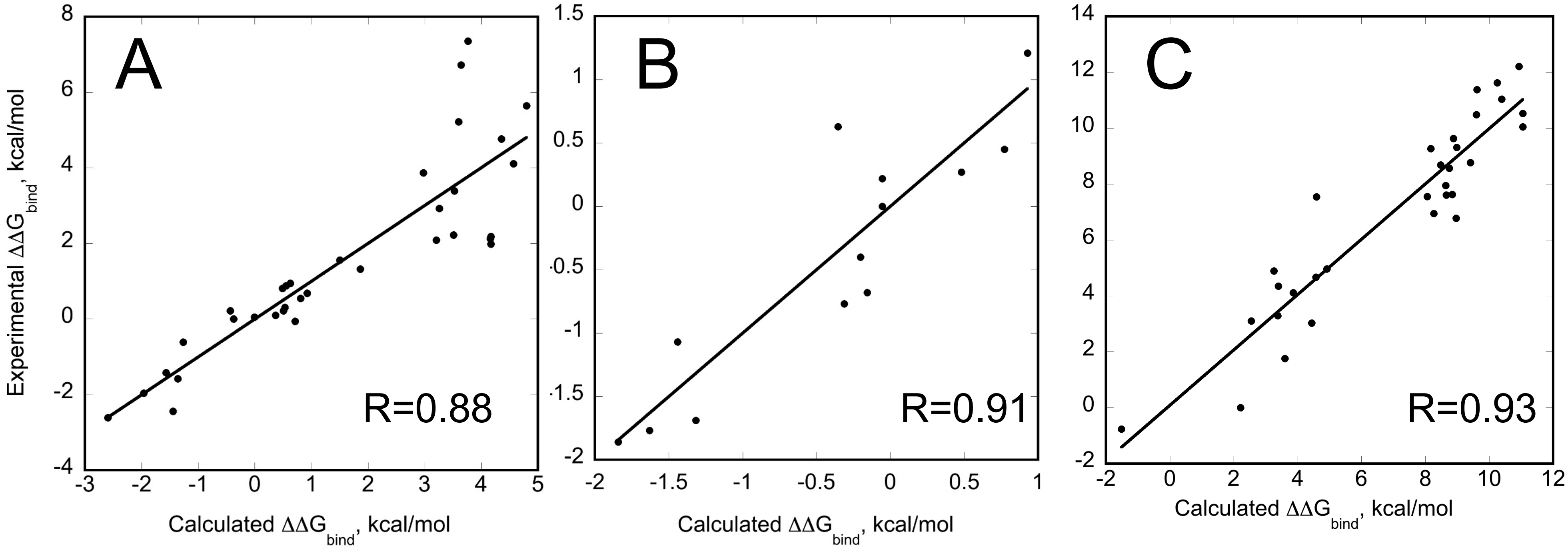

Figure S7: Correlation between $\Delta\Delta G_{bind}$ predicted from NGS and experimental $\Delta\Delta G_{bind}$ measured on purified proteins. (A) BPTI/ChT interactions; (B) BPTI/MT interactions; (C) BPTI/BT interactions. The best normalization formula was obtained that converts the enrichment value from 4 gates into the $\Delta\Delta G_{bind}$ value for each protease. The correlation coefficient R is shown on the graph. (C) is in modified from Heyne, M.; Papo, N.; Shifman, J. M., Generating quantitative binding landscapes through fractional binding selections combined with deep sequencing and data normalization. *Nat Commun* **2020,** 11(1), 1-7 under the terms of the CC BY license (Creative Commons Attribution 4.0 International License), Copyright © 2020, The Author(s)

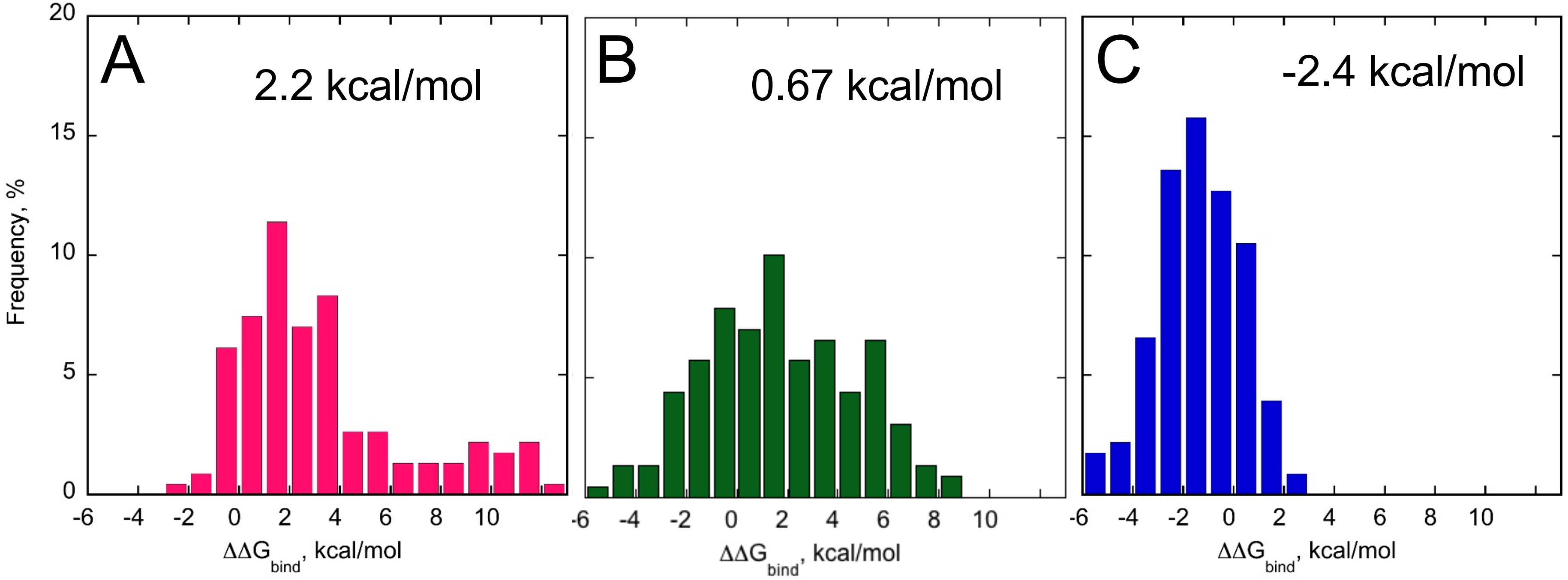

Figure S8: Histograms of $\Delta\Delta G_{bind}$ values due to single mutational steps for BT interacting with BPTI_K15R, BPTI_A16S and BPTI_K15A (A) BPTI_K15R mutation (B) BPTI_A16S mutation (C) BPTI_K15A mutation. Mean value for $\Delta\Delta G_{bind}$ for each histogram is displayed on each graph. 57%, 67%, 68% of all single mutational steps are summarized on the background of K15R, A16S, and K15A BPTI mutations, respectively.

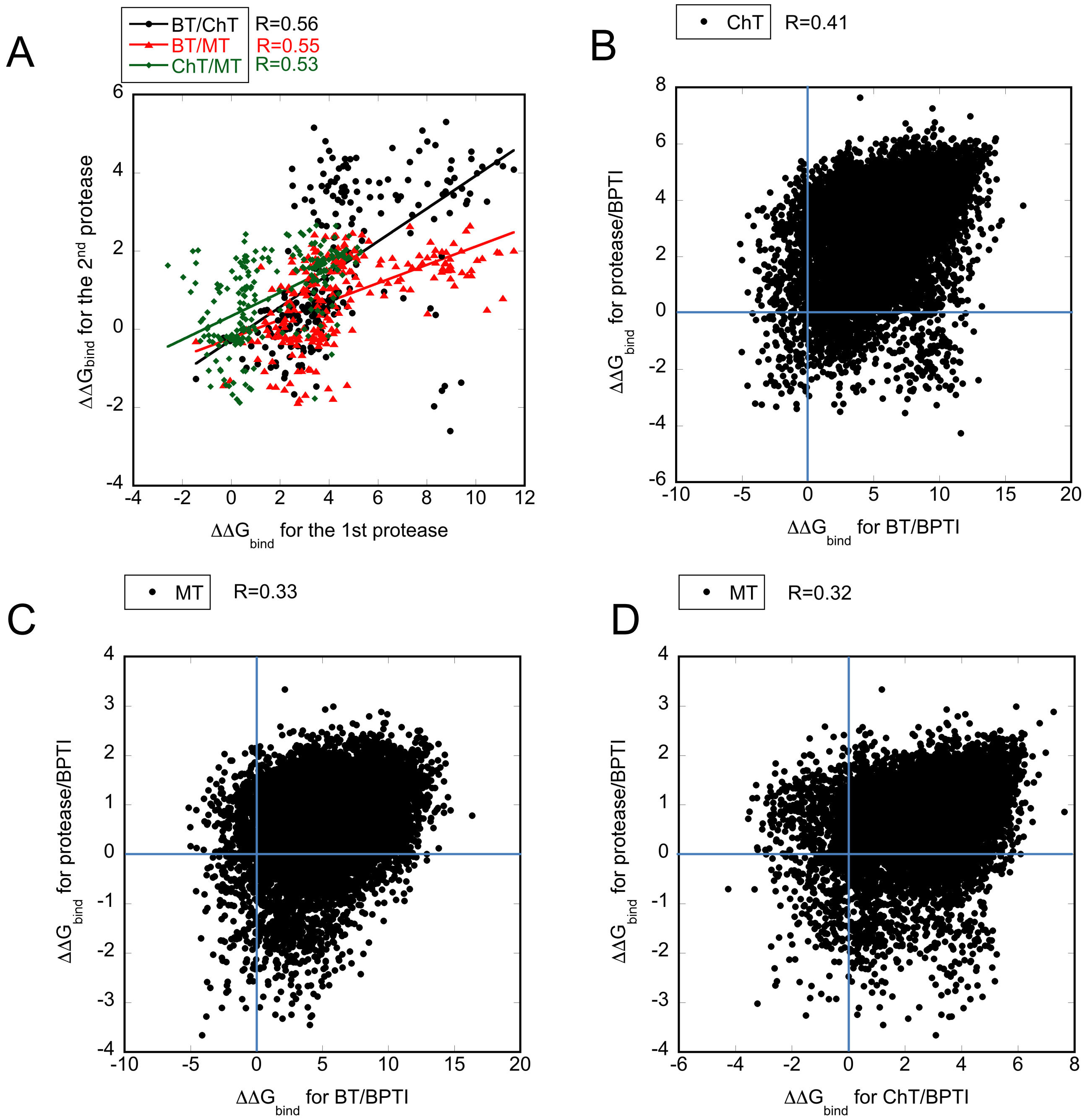

Figure S9: Pairwise correlations between $\Delta\Delta G_{bind}$ values for the same mutations in different PPIs. (A) shows the correlation for single mutations only. (B), (C), (D) shows correlation for all detected single and double mutations for BT vs. ChT (B), BT vs. MT (C) and ChT vs. MT (D). Blue lines separate the quadrants with different kinds of mutations. Right upper quadrant contains mutations with decreased affinity to both proteases, left bottom quadrant shows mutations with increased affinity to both proteases. Upper left and lower right quadrant contain specificity-enhancing mutations.

A

12	13	15	16	17	18	34	35	36	37	39	
3.9	3.5	3.7	3.6	3.2	3.4	2.8	2.8	3.6	2.9	2.3	**11**
	3.3	6.4	4.1	2.9	2.5	1.8	3.0	3.1	3.2	2.9	**12**
		4.8	4.0	3.4	3.5	2.9	2.6	3.4	3.5	3.1	**13**
			7.5	4.7	4.6	3.5	5.7	6.1	6.1	4.7	**15**
				4.2	4.2	3.5	4.6	4.8	4.6	3.5	**16**
					2.9	2.5	2.9	3.7	3.3	2.7	**17**
						2.5	2.7	3.0	2.5	2.6	**18**
							2.6	3.4	2.5	2.6	**34**
								4.2	3.4	3.6	**35**
									5.9	3.4	**36**
										3.1	**37**

B

12	13	15	16	17	18	34	35	36	37	39	
1.3	1.1	1.0	1.0	1.1	1.1	0.6	1.3	1.1	1.2	0.9	**11**
	1.2	2.7	3.3	1.8	1.5	1.1	2.3	2.9	2.2	1.1	**12**
		1.2	1.1	1.0	1.1	0.7	1.6	1.1	1.4	0.8	**13**
			2.7	1.8	1.4	0.9	2.0	2.3	1.8	0.9	**15**
				1.9	1.6	1.0	2.2	3.2	2.5	1.2	**16**
					1.4	1.0	1.7	1.9	1.7	1.0	**17**
						1.1	1.8	1.4	1.5	1.0	**18**
							1.5	1.2	1.5	1.0	**34**
								2.7	2.1	1.5	**35**
									3.0	1.4	**36**
										1.3	**37**

C

12	13	15	16	17	18	34	35	36	37	39	
1.4	0.7	1.4	1.3	0.7	1.1	0.8	1.5	1.7	1.0	0.8	**11**
	1.0	1.9	1.9	1.0	1.3	1.1	1.8	2.1	1.5	1.0	**12**
		1.2	1.0	0.7	0.8	0.7	1.2	1.3	1.1	0.7	**13**
			2.3	0.9	1.7	1.1	2.3	2.6	1.8	1.3	**15**
				1.0	1.7	1.1	2.3	2.5	1.7	1.0	**16**
					0.9	0.9	1.0	1.1	0.9	0.6	**17**
						0.9	1.8	1.8	1.3	0.9	**18**
							1.2	1.2	1.2	0.8	**34**
								2.7	1.8	1.3	**35**
									2.3	1.5	**36**
										1.0	**37**

Figure S10: The absolute values of coupling energies ΔGi averaged over a pair of BPTI positions: (A) BT/BPTI interaction; (B) ChT/BPTI interaction; (C) MT/BPTI interaction. On the top and on the right BPTI positions are shown. The values are color coded from large (black) to zero (white) and the average value is indicated in each square.

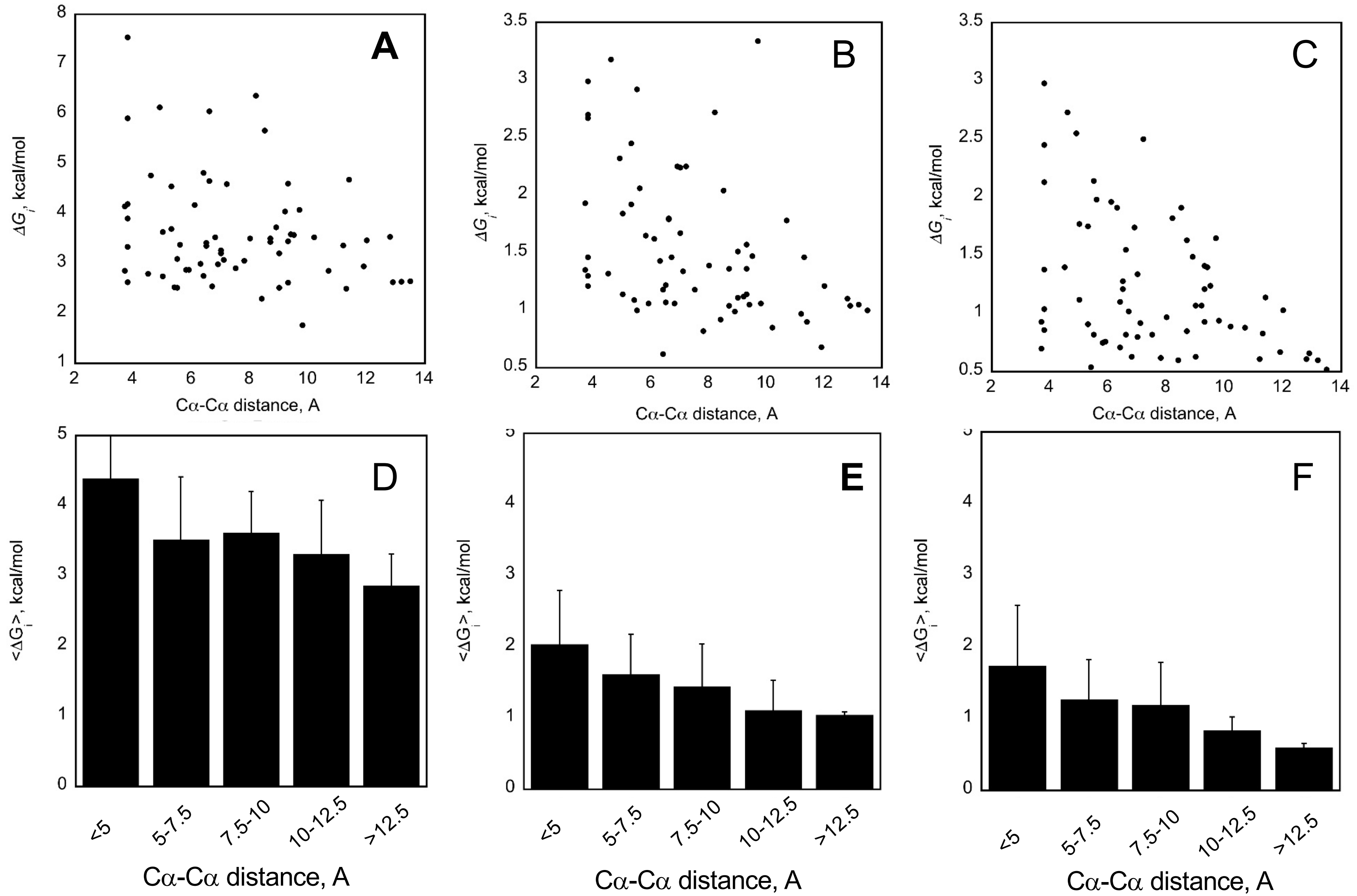

Figure S11: Dependence of ΔGi on the Cα-Cα distance between the two positions. Top panel shows dots that represent |ΔGi| for each pair of positions averaged over all recorded double mutations at these pair of positions. Bottom panel assigns |ΔGi| according to distance between the two Cα atoms and averages over each bin. Standard deviation for each average is shown. (A) and (D): BT/BPTI interaction; (B) and (E): ChT/BPTI interaction; (C) and (F): MT/BPTI interaction.

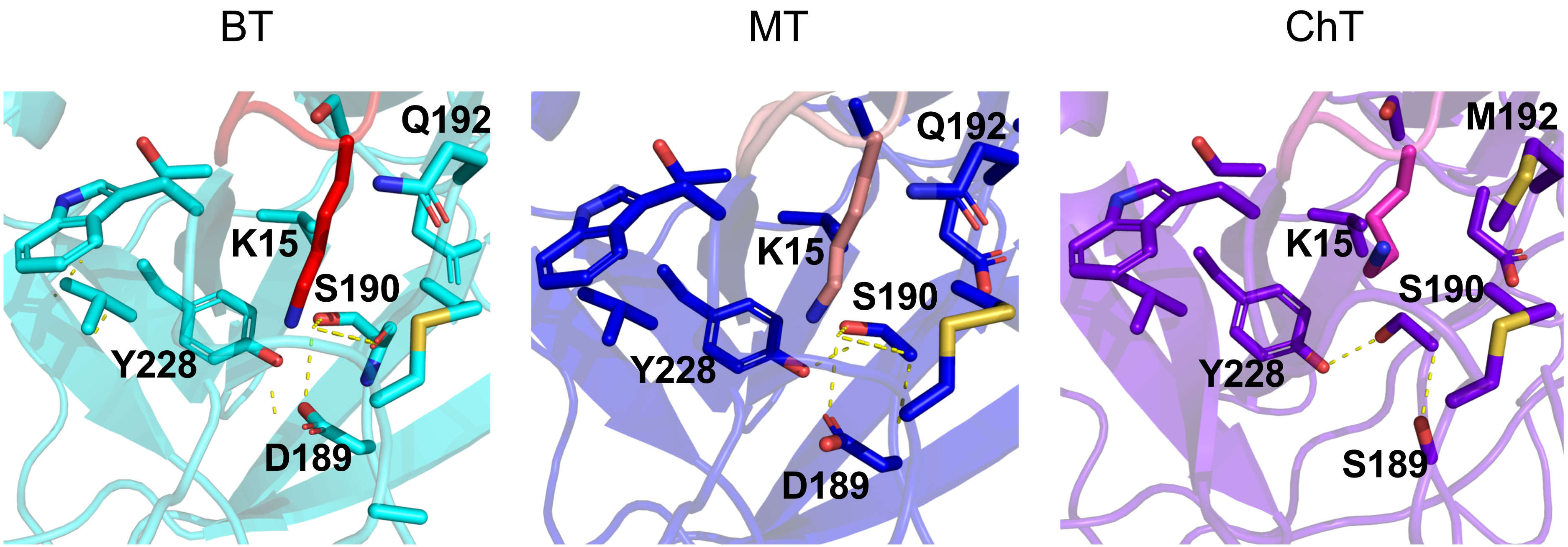

Figure S12: Structural comparison of the K15 binding site in the 3 proteases, BT (cyan), MT (dark blue), and ChT (violet). The K15 binding site is identical for BT and MT with K15 participating in a network of hydrogen bonds with Y228, D189, and S190. in ChT, D189 is substituted by a Ser, the hydrogen bond network with K15 is destroyed and K15 is pointing upwards toward a more solvent accessible area.

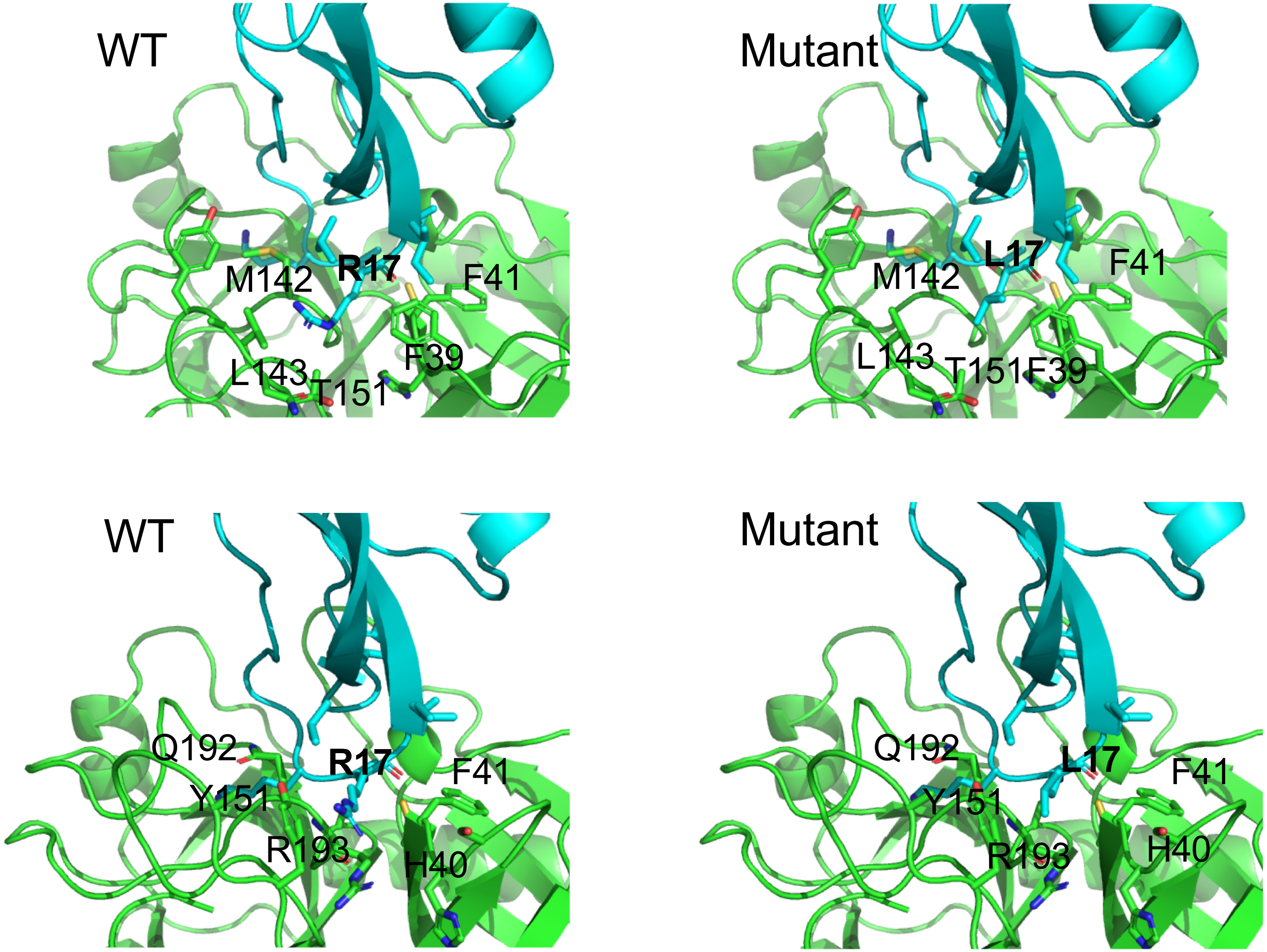

Figure S13: Position 17

Top: Structural comparison of interactions of ChT with BPTI WT(left) and BPTI R17L mutant (right). Protease is shown in green and BPTI in cyan. Bottom: Structural comparison of interactions of MT with BPTI WT(left) and BPTI R17L mutant (right). Protease is shown in green and BPTI in cyan. All participating residues are labeled.

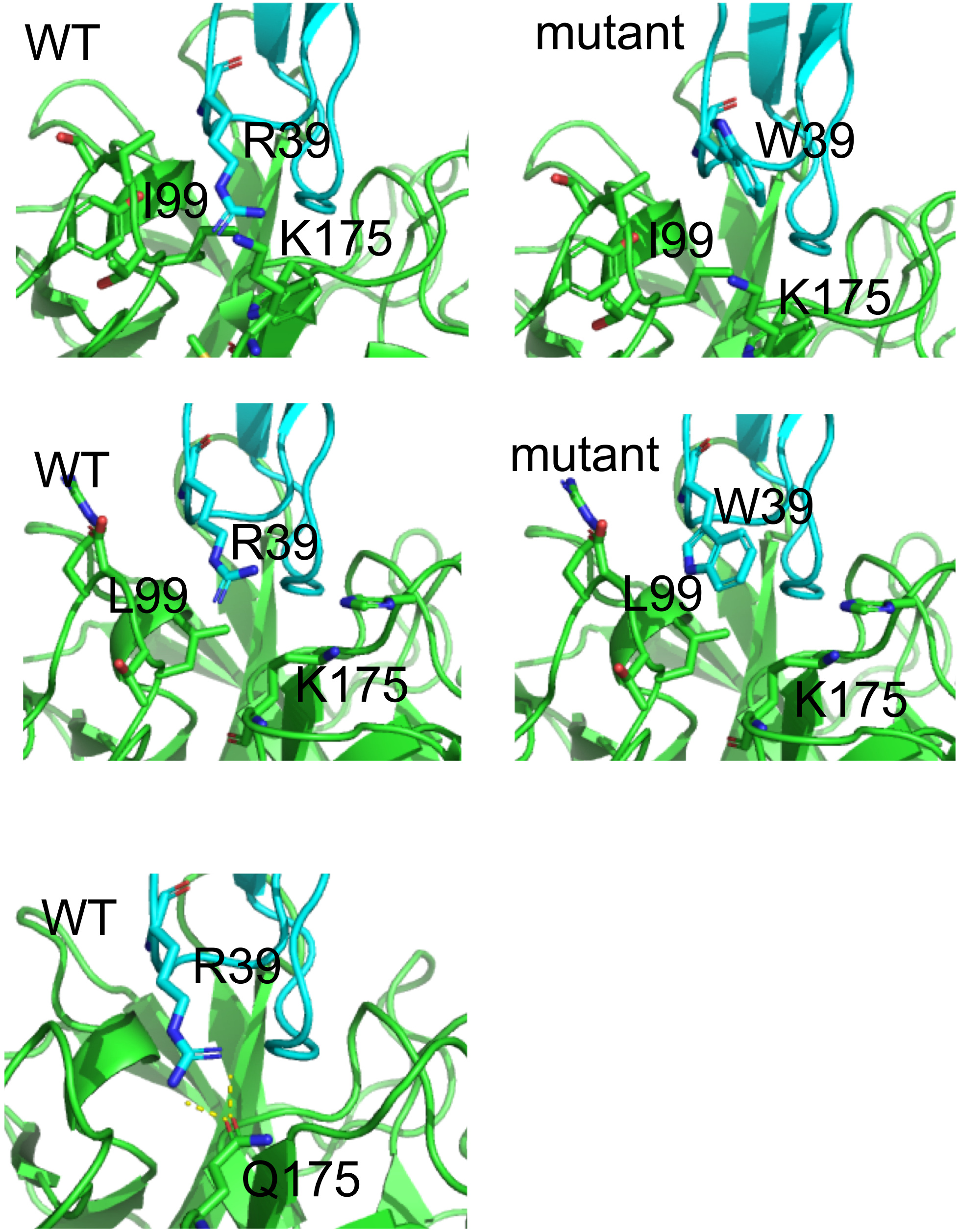

Figure S14: Position 39

Top: Structural comparison of interactions of ChT with BPTI WT(left) and BPTI R39W mutant (right). Protease is shown in green and BPTI in cyan. Middle: Structural comparison of interactions of MT with BPTI WT(left) and BPTI R39W mutant (right). Bottom: Interaction between WT BPTI and BT. Protease is shown in green and BPTI in cyan. All participating residues are labeled.

Supplementary Note 1

primers for construction of BPTI library

GGTGGTTCTGGTGGTGGTGGTTCTGGTGGTGGTGGTTCTGCTAGCAGACCAGATTTCTGCCTTGAA

11 12 13 15 16 17 18

CCACCATACACGGGTCCCTGCAAGGCAAGGATAATTAGGTACTTTTACAATGCGAAAGCAGGTCTGT

34 35 36 37 39

GCCAGACCTTCGTGTATGGGGGTTGCAGAGCCAAGAGAAATAACTTCAAGTCAGCTGAAGACTGCAT

GAGGACGTGCGGAGGCGCTGGATCCTTGCCAGATAAACCATTGGCTTTCCAAGATCCATCTGAACA

AAAGCTTATTTCTGAAGAGGACTTGTAATAGCTC

Reverse Complement:

GAGCTATTACAAGTCCTCTTCAGAAATAAGCTTTTGTTCAGATGGATCTTGGAAAGCCAATGGTTTATCTGGCAAG
GATCCAGCGCCTCCGCACGTCCTCATGCAGTCTTCAGCTGACTTGAAGTTATTTCTCTTGGCTCTGCAACCCCCATA
CACGAAGGTCTGGCACAGACCTGCTTTCGCATTGTAAAAGTACCTAATTATCCTTGCCTTGCAGGGACCCGTGTAT
GGTGGTTCAAGGCAGAAATCTGGTCTGCTAGCAGAACCACCACCACCAGAACCACCACCACCAGAACCACC

Legend

Green/Red Restriction site

Blue Positions intended to mutate

BPTI_TPCR_ FWD (forward) GATTTCTGCCTTGAACCACCATACAC (26 bases, 65°C)

BPTI_TPCR_R2 (reverse) CAGCTGACTTGAAGTTATTTCTCTTGGC (28 bases, 64°C)

BPTI11N2 (forward)
CAGATTTCTGCCTTGAACCACCATACNNSGGTCCCTGCAAGGCAAGGATAATTAG (26 bases, 65°C; 26 bases, 66°C)

BPTI_12N (forward) CTGCCTTGAACCACCATACACGNNSCCCTGCAAGGCAAGGATAATTAGG (22 bases, 63°C; 24 bases, 65°C)

BPTI_13N (forward) CCTTGAACCACCATACACGGGTNNSTGCAAGGCAAGGATAATTAGGTACTTTTAC (22 bases, 64°C; 30 bases 64°C)

BPTI_15N (forward) CACCATACACGGGTCCCTGCNNSGCAAGGATAATTAGGTACTTTTACAATGCG (20 bases, 64°C; 30 bases, 64°C)

BPTI_16N (forward) CCATACACGGGTCCCTGCAAGNNSAGGATAATTAGGTACTTTTACAATGCGAAAGCAG (21 bases, 66°C; 34 bases, 66°C)

BPTI_17N (forward) CGGGTCCCTGCAAGGCANNSATAATTAGGTACTTTTACAATGCGAAAGCAGG (17 bases, 65°C; 32 bases, 65°C)

BPTI18N2 (forward) GGGTCCCTGCAAGGCAAGGNNSATTAGGTACTTTTACAATGCGAAAGCAGGTC (19 bases, 66°C; 31 bases, 66°C)

BPTI34R2 (reverse) CTTGGCTCTGCAACCCCCATASNNGAAGGTCTGGCACAGACCTGC (21 bases, 65°C; 21 bases, 63°C)

BPTI_35R (reverse) CTCTTGGCTCTGCAACCCCCSNNCACGAAGGTCTGGCACAGACC (20 bases, 65°C; 21 bases, 64°C)

BPTI_36R (reverse) GAAGTTATTTCTCTTGGCTCTGCAACCSNNATACACGAAGGTCTGGCACAGACC (27 bases, 64°C; 24 bases, 64°C)

BPTI_37R (reverse) GACTTGAAGTTATTTCTCTTGGCTCTGCASNNCCCATACACGAAGGTCTGGCAC (29 bases, 66°C; 22 bases, 65°C)

BPTI39R2 (reverse) CAGCTGACTTGAAGTTATTTCTCTTGGCSNNGCAACCCCCATACACGAAGGTC (28 bases, 64°C; 22 bases, 66°C)

BPTI11.12N CAGATTTCTGCCTTGAACCACCATACNNSNNSCCCTGCAAGGCAAGGATAATTAGG (26 bases, 65°C; 24 bases, 65°C)

BPTI_11.13 (forward) CAGATTTCTGCCTTGAACCACCATACNNSGGTNNSTGCAAGGCAAGGATAATTAGGTACTTTTAC (26 bases, 65°C; 30 bases, 64°C)

BPTI_11.15 (forward) CAGATTTCTGCCTTGAACCACCATACNNSGGTCCCTGCNNSGCAAGGATAATTAGGTACTTTTACAATGCG (26 bases, 65°C; 30 bases, 64°C)

BPTI_11.16 (forward) CAGATTTCTGCCTTGAACCACCATACNNSGGTCCCTGCAAGNNSAGGATAATTAGGTACTTTTACAATGCGAAAGCAG (26 bases, 65°C; 34 bases, 66°C)

BPTI_11.17 (forward) CAGATTTCTGCCTTGAACCACCATACNNSGGTCCCTGCAAGGCANNSATAATTAGGTACTTTTACAATGCGAAAGCAGG (26 bases, 65°C; 32 bases, 65°C)

BPTI11.18N (forward) CAGATTTCTGCCTTGAACCACCATACNNSGGTCCCTGCAAGGCAAGGNNSATTAGGTACTTTTACAATGCGAAAGCAGGTC (26 bases, 65°C; 31 bases, 66°C)

BPTI_12.13 (forward) CTGCCTTGAACCACCATACACGNNSNNSTGCAAGGCAAGGATAATTAGGTACTTTTAC (22 bases, 63°C; 30 bases, 64°C)

BPTI_12.15 (forward) CTGCCTTGAACCACCATACACGNNSCCCTGCNNSGCAAGGATAATTAGGTACTTTTACAATGCG (22 bases, 63°C; 30 bases, 64°C)

BPTI_12.16 (forward)
CTGCCTTGAACCACCATACACGNNSCCCTGCAAGNNSAGGATAATTAGGTACTTTTACAATGCGAAAGCAG (22 bases, 63°C; 34 bases, 66°C)

BPTI_12.17 (forward)
CTGCCTTGAACCACCATACACGNNSCCCTGCAAGGCANNSATAATTAGGTACTTTTACAATGCGAAAGCAGG (22 bases, 63°C; 32 bases, 65°C)

BPTI_12.18 (forward)
CTGCCTTGAACCACCATACACGNNSCCCTGCAAGGCAAGGNNSATTAGGTACTTTTACAATGCGAAAGCAGGTC (22 bases, 63°C; 31 bases, 66°C)

BPTI_13.15 (forward)
CCTTGAACCACCATACACGGGTNNSTGCNNSGCAAGGATAATTAGGTACTTTTACAATGCG (22 bases, 64°C; 30 bases, 64°C)

BPTI_13.16 (forward)
CCTTGAACCACCATACACGGGTNNSTGCAAGNNSAGGATAATTAGGTACTTTTACAATGCGAAAGCAG (22 bases, 64°C; 34 bases, 66°C)

BPTI_13.17 (forward)
CCTTGAACCACCATACACGGGTNNSTGCAAGGCANNSATAATTAGGTACTTTTACAATGCGAAAGCAGG (22 bases, 64°C; 32 bases, 65°C)

BPTI_13.18 (forward)
CCTTGAACCACCATACACGGGTNNSTGCAAGGCAAGGNNSATTAGGTACTTTTACAATGCGAAAGCAGGTC (22 bases, 64°C; 31 bases, 66°C)

BPTI_15.16 (forward)
CACCATACACGGGTCCCTGCNNSNNSAGGATAATTAGGTACTTTTACAATGCGAAAGCAG (20 bases, 64°C; 34 bases, 66°C)

BPTI_15.17 (forward)
CACCATACACGGGTCCCTGCNNSGCANNSATAATTAGGTACTTTTACAATGCGAAAGCAGG (20 bases, 64°C; 32 bases, 65°C)

BPTI_15.18 (forward)
CACCATACACGGGTCCCTGCNNSGCAAGGNNSATTAGGTACTTTTACAATGCGAAAGCAGGTC (20 bases, 64°C; 31 bases, 66°C)

BPTI_16.17 (forward) CCATACACGGGTCCCTGCAAGNNSNNSATAATTAGGTACTTTTACAATGCGAAAGCAGG (21 bases, 66°C; 32 bases, 65°C)

BPTI_16.18 (forward)
CCATACACGGGTCCCTGCAAGNNSAGGNNSATTAGGTACTTTTACAATGCGAAAGCAGGTC (21 bases, 66°C; 31 bases, 66°C)

BPTI_17.18 (forward) CGGGTCCCTGCAAGGCANNSNNSATTAGGTACTTTTACAATGCGAAAGCAGGTC (17 bases, 65°C; 31 bases, 66°C)

BPTI_34.35 (reverse) CTCTTGGCTCTGCAACCCCCSNNSNNGAAGGTCTGGCACAGACCTGC (20 bases, 65°C; 21 bases, 63°C)

BPTI_34.36 (reverse) GAAGTTATTTCTCTTGGCTCTGCAACCSNNATASNNGAAGGTCTGGCACAGACCTGC (27 bases, 64°C; 21 bases, 63°C)

BPTI_34.37 (reverse) GACTTGAAGTTATTTCTCTTGGCTCTGCASNNCCCATASNNGAAGGTCTGGCACAGACCTGC (29 bases, 66°C; 21 bases, 63°C)

BPTI_34.39 (reverse) CAGCTGACTTGAAGTTATTTCTCTTGGCSNNGCAACCCCCATASNNGAAGGTCTGGCACAGACCTGC (28 bases, 64°C; 21 bases, 63°C)

BPTI_35.36 (reverse) GAAGTTATTTCTCTTGGCTCTGCAACCSNNSNNCACGAAGGTCTGGCACAGACC (27 bases, 64°C; 21 bases, 64°C)

BPTI_35.37 (reverse) GACTTGAAGTTATTTCTCTTGGCTCTGCASNNCCCSNNCACGAAGGTCTGGCACAGACC (29 bases, 66°C; 21 bases, 64°C)

BPTI_35.39 (reverse) CAGCTGACTTGAAGTTATTTCTCTTGGCSNNGCAACCCCCSNNCACGAAGGTCTGGCACAGACC (28 bases, 64°C; 21 bases, 64°C)

BPTI_36.37 (reverse) GACTTGAAGTTATTTCTCTTGGCTCTGCASNNSNNATACACGAAGGTCTGGCACAGACC (29 bases, 66°C; 24 bases, 64°C)

BPTI_36.39 (reverse) CAGCTGACTTGAAGTTATTTCTCTTGGCSNNGCAACCSNNATACACGAAGGTCTGGCACAGACC (28 bases, 64°C; 24 bases, 64°C)

BPTI_37.39 (reverse) CAGCTGACTTGAAGTTATTTCTCTTGGCSNNGCASNNCCCATACACGAAGGTCTGGCAC (28 bases, 64°C; 22 bases, 65°C)

The Tm was calculated on this website: http://tmcalculator.neb.com/#!/. Phusion Hot Start Flex (HF Buffer), 20 nM primer concentration.
